# Data-driven trial design: use of target trial emulation to evaluate eligibility criteria in asthma and COPD

**DOI:** 10.3389/fmed.2026.1863026

**Published:** 2026-06-23

**Authors:** Solomon B. Makgoeng, Margaret Gamalo, Leo J. Russo, Vivek Pradhan, Abhishek Bhattacharjee, Nandini Bhosale

**Affiliations:** 1 Pfizer Inc, Collegeville, PA, United States; 2 Pfizer Inc, Cambridge, MA, United States; 3 Pfizer Healthcare India Pvt. Ltd, Chennai, Tamil Nadu, India

**Keywords:** asthma, chronic obstructive pulmonary disease, dupilumab, eligibility criteria, modified Trial Pathfinder, real-world data, trial emulation

## Abstract

**Background:**

Clinical trial eligibility criteria are crucial to ensuring valid estimation of therapeutic treatment effects and robust measurement of safety. However, highly restrictive criteria have a negative impact on study generalizability and patient accrual.

**Methods:**

Following the target trial emulation framework, we applied a modified Trial Pathfinder approach to emulate dupilumab trials for chronic obstructive pulmonary disease (COPD; NCT03930732) and asthma (NCT02414854), using Optum Market Clarity electronic health records and annualized exacerbation rate ratio (AERR) as the primary endpoint. We attempted to match the rigor of the original trial effect estimates using doubly robust, augmented inverse probability-weighted estimates for AERR and corresponding confidence intervals using the bootstrap method. We computed *E*-values to assess the potential impact of unmeasured confounding and applied empirical assessments of improvement in the generalizability of the study population under data-driven eligibility criteria.

**Results:**

We identified dupilumab new-user cohorts for COPD (*N* = 388,051) and asthma (*N* = 915,154) in electronic health records. The number of original criteria successfully emulated was 29 (out of 47) for COPD and 21 (out of 38) for asthma. After application of all emulated eligibility criteria, cohort sample sizes were markedly reduced (COPD: *N* = 4,333; asthma: *N* = 115,761). Analysis with Shapley values retained nine criteria for COPD and 10 for asthma. After applying this data-driven set of criteria, cohort sample sizes increased (COPD: *N* = 118,739; asthma: *N* = 219,580) while AERR point estimates changed by less than 10% with a narrower confidence interval width.

**Conclusion:**

Our findings demonstrate the use of trial emulation and Trial Pathfinder for data-driven identification of eligibility criteria for relaxation or removal in non-oncology therapeutic areas. Thorough assessment for unmeasured confounding is essential for credible causal inference. Lastly, the comparison of sample characteristics provided valuable insights into how data-driven eligibility criteria enhance the generalizability of clinical trials.

## 1 Introduction

Clinical trial emulation using real-world data (RWD) is rapidly expanding. Key applications of trial emulation include (1) a framework for designing observation studies, when a randomized trial is not feasible or ethical, and causal inferences need to be drawn from observational data, and (2) design of a future trial, where emulation is used to evaluate aspects of the trial design. Trial emulation can aid decisions on endpoints, length of follow-up, treatment strategies, and eligibility criteria (EC) (1, 2).

EC are crucial in clinical trials because they determine the characteristics of the target population for which a medical intervention can be indicated. Professional medical organizations across therapeutic areas have given attention to the need to modernize the approach to setting trial criteria (3). Overly restrictive criteria can hinder patient accrual, reduce the generalizability of study results to the full target patient population that could benefit from the medical intervention under study, and decrease the empirical statistical power for a study to detect a true treatment effect (1–6).

Several approaches for evaluating the modifying effect of EC have been suggested in the literature (7–10). Liu et al. describe an approach that is easy to understand and implement, using a tool called Trial Pathfinder (11).

This study aims to build on prior applications (11, 12) of Trial Pathfinder to evaluate potential trial EC and add these approaches: (1) demonstrate the value of trial emulation for diseases and endpoints different from those examined previously (7–10), (2) assess unmeasured confounding to support robustness of inferences, and (3) illustrate measures of improvements in representativeness of the new trial population relative to the original, more restrictive sample. Previous research using Trial Pathfinder has focused heavily on oncology with a primary endpoint of survival (11, 12). We have extended the body of research by emulating trials for asthma and chronic obstructive pulmonary disorder (COPD) with non-mortality endpoints on the risk ratio scale.

## 2 Methods

### 2.1 Data sources

We used Optum Market Clarity electronic health record (EHR) data, which is a comprehensive EHR repository derived from several healthcare provider organizations across the US, to emulate the trials. The data source aggregates de-identified EHR data from more than 57 contributing systems and 111,000 sites of care. Optum EHR captures patient-level structured and unstructured data, including medical history, laboratory test results, and treatment records. Optum EHRs encompass over 82 million unique patient lives, featuring rich, longitudinal clinical variables (20,000+ mapped), more than 1 billion prescriptions, and data from 150+ US payers, and is part of the FDA Sentinel (13, 14).

### 2.2 Study design

#### 2.2.1 Cohort creation

We selected completed trials from ClinicalTrials.gov for dupilumab that resulted in market authorization for asthma and COPD. Dupilumab is a monoclonal antibody that inhibits interleukin 4 and interleukin 13 receptor signaling and is used for the treatment of allergic diseases such as atopic dermatitis, asthma, and nasal polyps, which result in chronic sinusitis (15).

**Figure 1:**
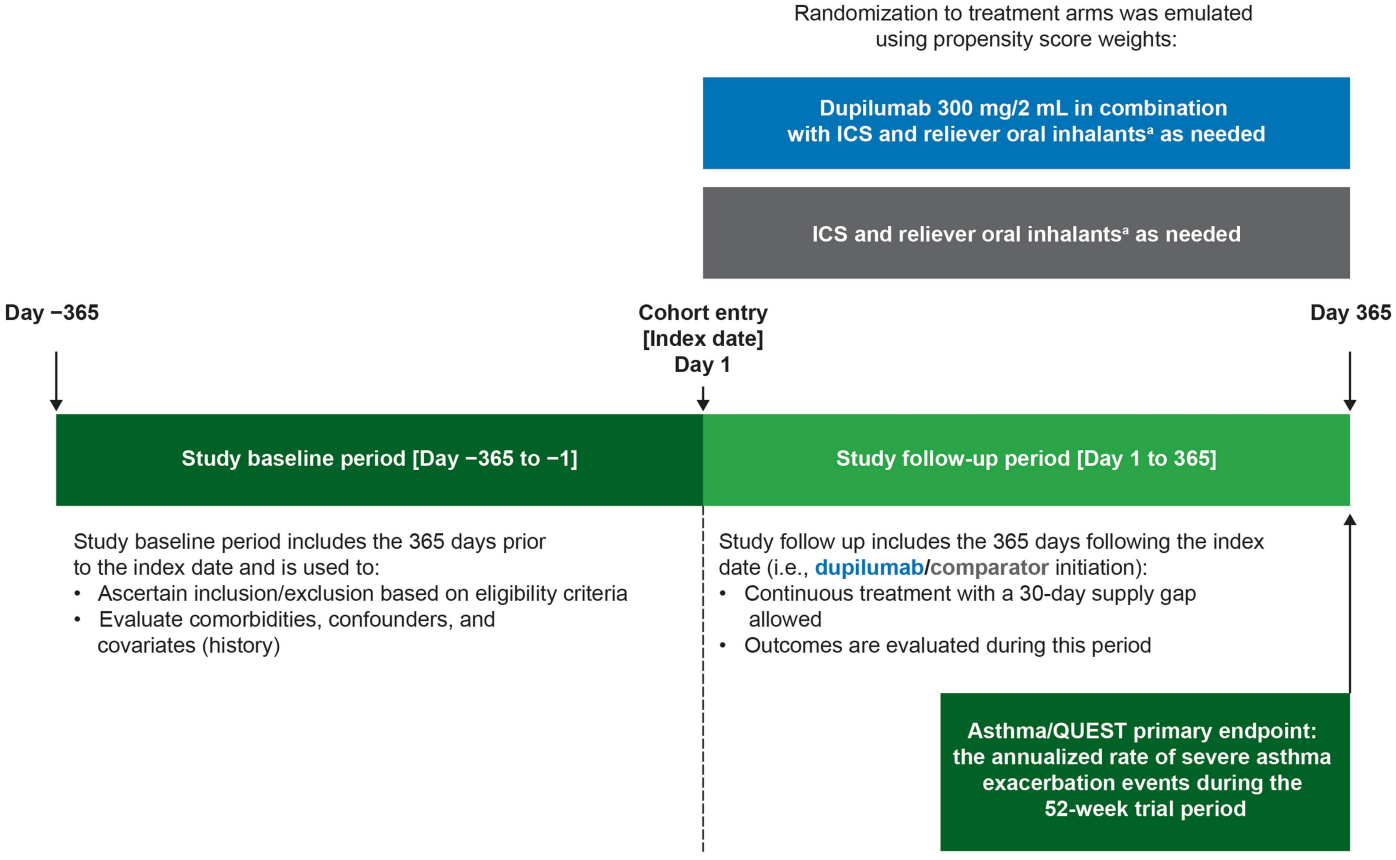
Asthma observational cohort study design for emulation of the Asthma/Quest trial (NCT02414854). Dark green represents the Pre-Index Period (1 year prior); light green represents the Post-Index Period (1 year after); and the index date is the start of treatment and follow-up. ^a^Reliever oral inhalants include albuterol/salbutamol levalbuterol/levosalbutamol. ICS, inhaled corticosteroid.

**Figure 2:**
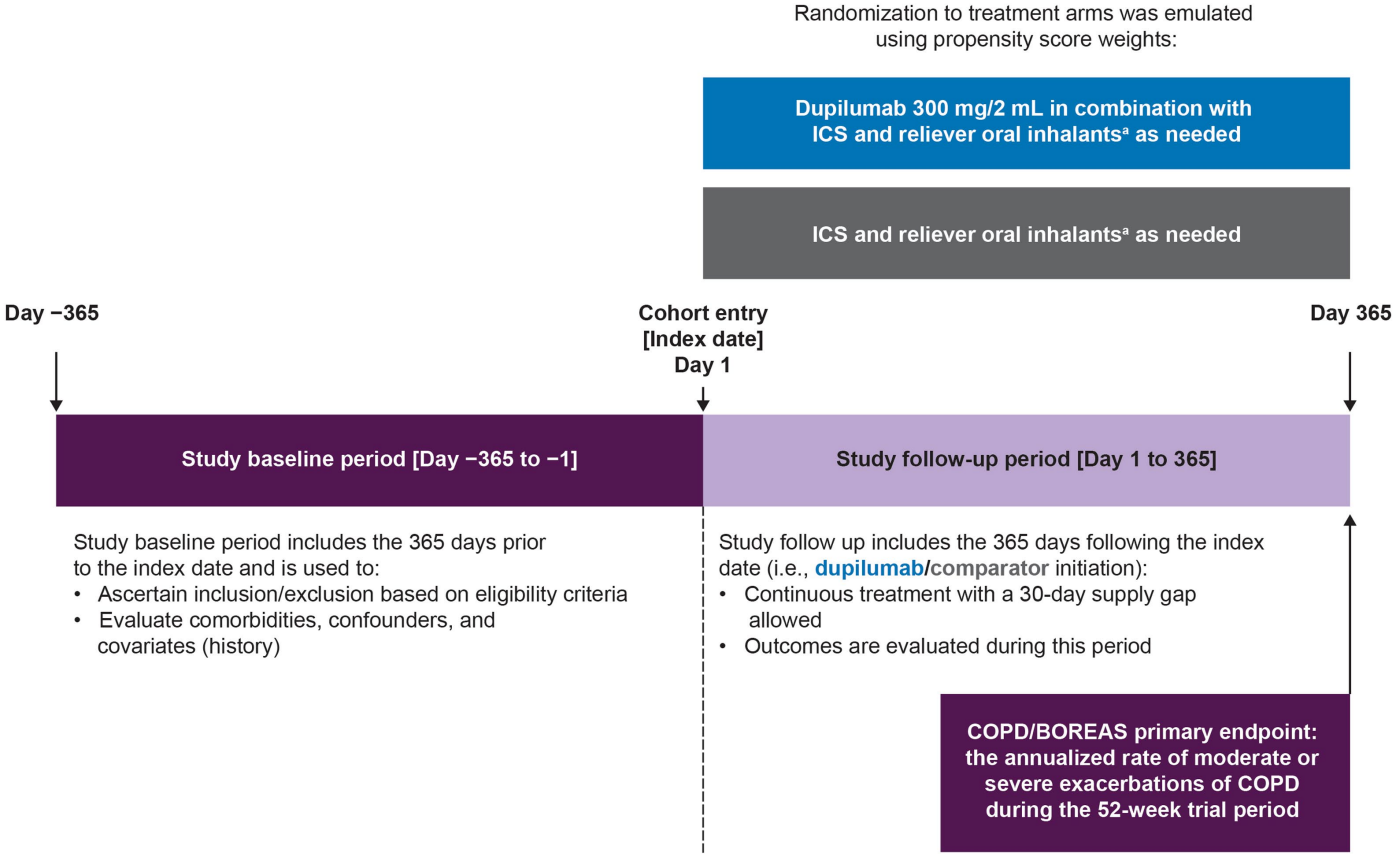
COPD observational cohort study design for emulation of the COPD/BOREAS trial (NCT03930732). Dark purple represents the Pre-Index Period (1 year prior); light purple represents the Post-Index Period (1 year after); and the index date is the start of treatment and follow-up. ^a^Reliever oral inhalants include albuterol/salbutamol levalbuterol/levosalbutamol. COPD, chronic obstructive pulmonary disease; ICS, inhaled corticosteroid.

The approval date of dupilumab for asthma (2018) and COPD (2024) allowed for adequate accrual of RWD records for routine care patients using the therapy at approved doses. We applied a previously published Target Trial Framework to replicate the full published protocol of the COPD/BOREAS (NCT03930732) and Asthma/Quest (NCT02414854) trials in Optum EHR (16–18). We applied a new-user cohort design for each of the two trial emulations to compare initiators of dupilumab at approved doses, with initiators of the standard of care treatment as comparator patients. We set cohort entry (i.e., index date) as the day of dupilumab or standard of care treatment initiation (Figures 1, 2). Patients were required to have evidence of database activity in the 12 months prior to cohort entry (drug exposure). Details of RWD cohort selection for each trial emulation, including details of diagnosis codes to identify relevant patients and emulation of trial EC in Optum EHR, are available in Supplementary Tables S1 and S2.

#### 2.2.2 Emulation of treatment arms and endpoints

We emulated the active arms for both the COPD/BOREAS and Asthma/QUEST trials with dupilumab 300 mg/2 mL in combination with standard-of-care inhaled corticosteroid (ICS) therapy and reliever inhalers as needed (i.e., albuterol/salbutamol levalbuterol/levosalbutamol) (Figures 1, 2). Comparator arms were replicated as ICS therapy, and reliever oral inhalants as needed as needed (i.e., albuterol/salbutamol levalbuterol/levosalbutamol), to mimic the trial comparator arms that differed only in that they included a subcutaneous placebo injection in addition to these treatments.

The COPD/BOREAS and Asthma/Quest trials both defined their primary endpoint as the annualized exacerbation rate of moderate/severe COPD/asthma events during a 52-week trial period. Real-world evidence (RWE) outcomes corresponding to these endpoints were defined as a deterioration of COPD or asthma symptoms requiring use of systemic corticosteroids for ≥3 days for COPD in the outpatient setting or hospitalization or emergency room visit with COPD or asthma as the primary diagnosis and requiring systemic corticosteroids for any duration during the hospitalization encounter.

### 2.3 Data analysis

#### 2.3.1 Emulation of randomization with propensity score weights

For each trial, we emulated randomization to treatment arms using propensity score (PS) weights (inverse probability of treatment weighting and augmented inverse probability of treatment weighting) to control for potential confounders measured during the 12-month baseline period prior to cohort entry. RWE emulation of randomization via PS weighting was based on a relevant set of baseline covariates to balance a credible set of potential confounders or their proxies. The list of baseline covariates included in the PS model is available in Supplementary Table S3.

#### 2.3.2 Implementation of modified Trial Pathfinder and treatment effect estimation

Each treatment effect was estimated with PS-weighted annualized exacerbation rate ratio (AERR) to provide treatment effect estimates that are robust to confounding (further details are provided in the Supplementary material) (19). For each trial, we implemented the modified Trial Pathfinder to quantify the change in AERR of exacerbation events resulting from exclusion of each EC averaged across all possible combinations of criteria subsets using Shapley values. Shapley values close to 0 suggest that a given criterion does not have a significant effect on the AERR treatment effect estimate, while negative Shapley values indicate that applying the criterion decreases AERR, demonstrating a better treatment effect for active treatment. Conversely, positive Shapely values identify EC that increase AERR. Trial Pathfinder defines EC to be retained if the Shapley value is less than 0 to improve the efficacy to be detected versus causing it to move toward the null (i.e., AERR = 1.00), as represented by Shapley values greater than 0. Because the measure of effect is efficacy, it is desirable to reduce its magnitude away from 1.00 (12).

We also implemented the bootstrap method of our analyses to ensure robust estimates of uncertainty intervals for Shapley point estimates. For each emulated trial, we generated 1,000 bootstrap samples with replacement. We then applied the modified Trial Pathfinder algorithm to each bootstrapped dataset to generate 1,000 Shapley value estimates, using the quantiles of the resulting distribution to derive the point estimate and bootstrap confidence interval (CI).

#### 2.3.3 Evaluating unmeasured confounding and representativeness

Lastly, we calculated *E*-values to demonstrate the robustness to unmeasured confounding effects for our findings on optimal data-driven sets of EC (20). We also demonstrated the representativeness improvement by calculating Generalizability Index for Study Traits (GIST) 2.0 measures for key demographic characteristics that are relevant to the FDA draft guidance on meaningful representation in underserved populations (21, 22). Distributions of baseline covariates were compared between the sampling frame (no randomized controlled trial criteria applied), the emulated trial with data-driven criteria, and the emulated trial with all criteria.

## 3 Results

The emulated EC and changes in cohort sizes when applied to asthma and COPD, respectively, are presented in Tables 1, 2. Patient counts show which EC would exclude or include large numbers of future trial patients.

**Table 1:** Asthma cohort: emulated trial criteria and resulting patient counts from Optum EHR.

Category	Selection/eligibility criterion	Comparator	Dupilumab
Sampling frame criteria	Starting population	993,427	11,041
	Received any asthma treatment	993,428	11,041
	Database activity in baseline	993,429	11,041
Study sample (emulated trial) inclusion criteria	Age ≥12	883,179	10,348
	Asthma exacerbation history	397,706	5,927
Study sample (emulated trial) exclusion criteria	Weight kg ≤ 30	841,218	10,075
	Lung disease baseline	884,877	9,179
	Current smoker	689,577	8,471
	Any asthma exacerbation 1 month pre-index	956,115	10,053
	Death baseline	992,122	11,039
	RTI 1 month pre-index	937,945	10,636
	Alcohol use in baseline	967,083	10,847
	Beta blockers (non-selective) in baseline	990,686	11,013
	Allergen immunotherapy in baseline	993,424	11,040
	Bronchial thermoplasty in baseline	993,393	11,032
	Prohibited concomitant therapy in baseline	956,614	10,065
	Pregnancy in baseline	940,321	10,568
	Parasitic infection in baseline	993,342	11,041
	HIV in baseline	989,703	11,007
	Infection baseline	834,932	9,213
	Live vaccination 1 month pre-index	991,739	11,036
	Malignancy	940,381	10,527
	Hepatitis in baseline	986,673	11,005
	Hepatobiliary disease or ALTE36 in baseline	982,297	10,985
	Abnormal lab values 1 month pre-index	981,476	11,003

ALTE36, alanine transaminase equals 36 units/liter; EHR, electronic health record; HIV, human immunodeficiency virus; ICS, inhaled corticosteroid; RTI, respiratory tract infection.

**Table 2:** COPD cohort: emulated trial criteria and resulting patient counts from Optum EHR.

Category	Selection/eligibility criterion	Comparator	Dupilumab
Sampling frame criteria	Starting population	385,451	2,600
	Received any COPD treatment	385,451	2,600
	Database activity in baseline	385,451	2,600
Study sample (emulated trial) inclusion criteria	Age ≥40 or ≤80	322,267	2,246
	Smoking history	275,360	1,396
	Exacerbation history	122,289	1,305
	BMI ≥ 16 kg/m^2^	380,920	2,585
Study sample (emulated trial) exclusion criteria	COPD diagnosis duration before index date	219,434	1,293
	Asthma diagnosis	342,236	1,431
	Other pulmonary diseases	124,048	370
	Cardiac conditions	382,483	2,587
	Oxygen therapy	384,427	2,577
	Hypercapnia	370,987	2,522
	Recent exacerbation	347,810	2,161
	Respiratory infection	300,034	2,279
	Surgical history	385,442	2,600
	α-1 anti-trypsin deficiency	384,368	2,583
	Biologic therapy	384,994	2,523
	Recent cardiovascular events	361,585	2,529
	TIA or stroke	365,850	2,528
	Hospitalization	380,619	2,591
	Previous dupilumab use	385,361	2,496
	Pregnancy or lactation	375,568	2,490
	Parasitic infection	385,411	2,600
	HIV infection	383,558	2,588
	Acute or chronic infection	275,667	2,094
	Live vaccinations	385,173	2,599
	Autoimmune disease	371,133	2,445
	Hepatitis	378,070	2,565
	Laboratory abnormalities	314,090	2,411
	Macrolide therapy	385,230	2,600

BMI, body mass index; COPD, chronic obstructive pulmonary disease; EHR, electronic health record; HIV, human immunodeficiency virus; ICS, inhaled corticosteroid; TIA, transient ischemic attack.

We identified dupilumab new-user cohorts for COPD (*N* = 388,051) and asthma (*N* = 915,154) in Optum EHR using sampling frames that represent the target population of patients receiving routine clinical care. We emulated over half of the full EC for COPD/BOREAS (29 out 47 criteria) and Asthma/Quest (21 out of 38 criteria) trials (Table 3). After applying the emulated EC to these initial sampling frame cohorts, we applied PS weighting to balance potentially confounding baseline variables. Fully emulated trial cohort sample sizes were markedly reduced (*N* = 4,333 for COPD; *N* = 115,761 for asthma), indicating the restrictiveness of the full EC (Table 3). Treatment effect estimates were comparable between the original published results for the COPD/BOREAS and Asthma/Quest trials and our RWE emulations (Table 3).

**Table 3:** Comparing original trial results versus RWD emulations; count of EC, count of patients, effect estimates.

Disease	NCT number	Original trial			Emulated trial: no RCT criteria			Emulated trial: all criteria applied			Emulated trial: data-driven criteria		
		EC count	*N*	AERR (95% CI)	EC count	*N*	AERR (95% CI)	EC count	*N*	AERR (95% CI)	EC count	*N*	AERR (95% CI)
COPD	NCT03930732	47	939	0.77 (0.60, 0.99)	–	388,051	0.65 (0.63, 0.68)	29	4,333	0.51 (0.24, 0.79)	9	118,739	0.35 (0.27, 0.43)
Asthma	NCT02414854	38	954	0.54 (0.43, 0.68)	–	915,154	0.61 (0.57, 0.63)	21	115,761	0.48 (0.44, 0.52)	10	219,580	0.43 (0.41, 0.46)

AERR: the effect of dupilumab versus comparator on trial outcome (acute disease exacerbation). AERR, annualized exacerbation rate ratio; CI, confidence interval; COPD, chronic obstructive pulmonary disease; EC, eligibility criteria; *N*, number of patients; RCT, randomized controlled trial; RWD, real-world data.

**Figure 3:**
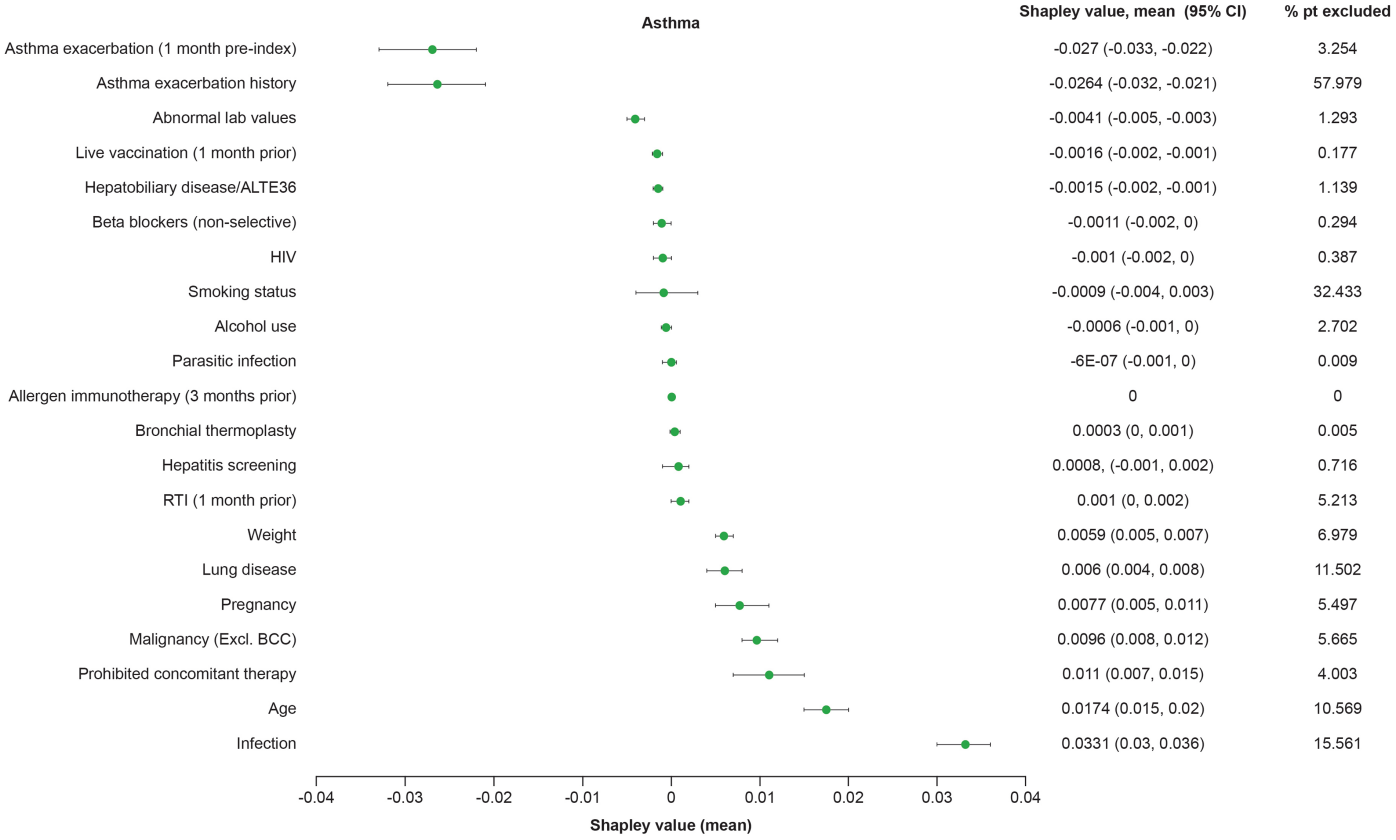
Asthma—mean Shapley values with 95% CI. This figure displays the mean Shapley values for each emulated eligibility criterion in the asthma cohort. Shapley values represent the average marginal contribution of each criterion to the treatment effect estimate (AERR) across all possible subsets of criteria. The mean values and their 95% CIs were calculated from 1,000 bootstrap samples to ensure robust estimation. Negative values (to the left of the zero line) indicate criteria that, on average, strengthen the treatment effect by lowering the AERR. Positive values indicate criteria that weaken the treatment effect. The magnitude of the value reflects the relative importance of that criterion. The “% pt. excluded” column quantifies the proportion of patients from the initial sampling frame who were removed by the application of each individual criterion. AERR, annualized exacerbation rate ratio; ALTE36, alanine transaminase equals 36 units/liter; BCC, basal cell carcinoma; CI, confidence interval; HIV, human immunodeficiency virus; pt., patient; RTI, respiratory tract infection.

**Figure 4:**
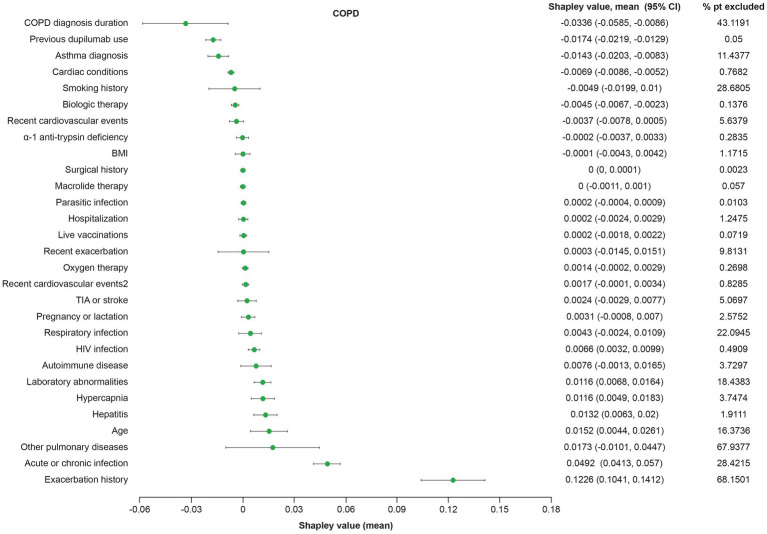
COPD—mean Shapley values with 95% CI. This figure displays the mean Shapley values for each emulated eligibility criterion in the COPD cohort. Shapley values represent the average marginal contribution of each criterion to the treatment effect estimate (AERR) across all possible subsets of criteria. The mean values and their 95% CIs were calculated from 1,000 bootstrap samples to ensure robust estimation. Negative values (to the left of the zero line) indicate criteria that, on average, strengthen the treatment effect by lowering the AERR. Positive values indicate criteria that weaken the treatment effect. The magnitude of the value reflects the relative importance of that criterion. The “% pt. excluded” column quantifies the proportion of patients from the initial sampling frame who were removed by the application of each individual criterion. AERR, annualized exacerbation rate ratio; BMI, body mass index; CI, confidence interval; COPD, chronic obstructive pulmonary disease; HIV, human immunodeficiency virus; pt., patient; TIA, transient ischemic attack.

Applying a modified Trial Pathfinder approach to determine optimal data-driven subsets (Shapley values less than 0) of the original EC of the COPD/BOREAS and Asthma/Quest trials, we found application of 10 criteria for asthma and 9 for COPD to be optimal in each case (Figures 3, 4). Cohort sample sizes for trial emulations with data-driven EC increased (*N* = 118,739 for COPD; *N* = 219,580 for asthma) while AERR point estimates changed by less than 10% from the original trials (16, 17) with narrower CI widths (Table 3). Additionally, AERR estimates and cohort sample sizes following the marginal application of EC are presented in Supplementary Tables S4 and S5 for asthma and COPD cohorts, respectively. Our *E*-value estimates for each treatment effect analysis found that unmeasured confounding effects would have to be very large to bias our treatment effect estimates and EC selection significantly. In the asthma and COPD data-driven trial emulations, for the effect estimate to be attributed to an unmeasured confounder, they would need to be associated with exposure and outcome at greater than 5-fold and 4-fold levels, respectively. This suggests that reduction of EC in the data-driven emulation did not increase unmeasured confounding (Table 4). Lastly, our calculations of the GIST 2.0 measure of representativeness of the distributions of key demographic variables of age, sex, race, and ethnicity suggest meaningful improvements in representativeness when applying the data-driven set of EC compared to when applying the original set of trial criteria (Table 5).

**Table 4:** Confounding bias assessment using *E*-values for determination of data-driven cohorts.

Disease	Cohort	AERR estimate (95% CI)	*E*-value estimate
COPD	Original	0.51 (0.24, 0.79)	3.33 (1.84)
	Fully relaxed	0.65 (0.63, 0.68)	2.40 (2.21)
	Data-driven	0.35 (0.27, 0.43)	5.16 (4.08)
Asthma	Original	0.48 (0.44, 0.52)	3.58 (3.25)
	Fully relaxed	0.61 (0.57, 0.63)	2.66 (2.50)
	Data-driven	0.43 (0.41, 0.46)	4.08 (3.77)

*E*-values quantify the minimum strength of association that an unmeasured confounder would need to have with both the exposure and the outcome to fully explain away the observed association (i.e., AERR). Higher *E*-values indicate greater robustness to potential unmeasured confounding. Values in parentheses represent the lower confidence limit of the *E*-value.

AERR, annualized exacerbation rate ratio; CI, confidence interval; COPD, chronic obstructive pulmonary disease.

**Table 5:** Assessment of improvement in study sample representation: comparing original and emulated trials with data-driven criteria to sampling frame for COPD and asthma.

Disease	Cohort	GIST
COPD	Original	0.122
	Data-driven	0.309
Asthma	Original	0.116
	Data-driven	0.579

GIST quantifies how well the study cohort represents the target population with respect to key study traits. Scores range from 0 to 1, where values closer to 1 indicate higher representativeness and greater generalizability. In this analysis, the target population was defined as the entire sampling frame, serving as the reference population. Thus, higher GIST scores indicate greater similarity of the study cohort to the overall population within the sampling frame, reflecting improved sample representativeness and the potential applicability of study findings to the broader population. COPD, chronic obstructive pulmonary disease; GIST, Generalizability Index for Study Traits.

## 4 Discussion

Designing trials with optimal, data-driven EC could address several current challenges of clinical research: patient pools could be larger, which should ease recruitment delays experienced by many trials, and trials could include more patients from underserved populations (1, 23). It has been shown that restrictive EC disproportionally excludes historically underrepresented groups (1). Increased representation of target populations may also aid in the translation of clinical trial findings to clinical care, which is a prominent challenge across many diseases (24, 25).

For both the COPD/BOREAS and Asthma/Quest trials, our data-driven RWE emulations resulted in comparable treatment effect estimates. Our analyses using *E*-values support that differences in treatment effect are due to effect modification and not unmeasured confounding.

In this paper, we addressed several key issues that are missing from current literature. Firstly, we demonstrate that the modified Trial Pathfinder approach can be successfully applied to non-oncology therapy areas with endpoints not as well defined as survival. We applied the approach to COPD/BOREAS and Asthma/Quest trial emulations, using a different measure of treatment effect (AERR), and still reached robust conclusions in evaluating EC relaxation in terms of increasing sample size and not modifying the treatment effect. This suggests that the modified Trial Pathfinder approach is generalizable to other therapeutic areas where the measure of treatment effect may be different.

Building on this demonstrated generalizability, the framework’s statistical core is inherently modular. This allows the same data-driven principles to be applied to a variety of common endpoint structures. For example, the impact of EC can be assessed on continuous outcomes, such as a change from a baseline measurement; on binary outcomes, like achieving a clinical response; and on time-to-event data to evaluate influences on survival. This adaptability ensures that the method is a robust tool for optimizing trial design across a wide spectrum of research questions, regardless of the specific data type of the primary endpoint.

From a practical standpoint, this approach is well suited for diseases that are captured with high accuracy in RWD, such as cardiovascular events, and treatments that have been on the market long enough for adequate utilization in routine care patients and could be suitable for emulation using our approach.

Furthermore, we demonstrated that assessment for unmeasured confounding impacts on the effects of EC contributes to the credibility of EC evaluation using modified Trial Pathfinder. This is in addition to comparability of treatment effects of the results from trial emulations to the original published results, demonstrating robustness against confounding biases bolsters the validity of results.

We provided representativeness metrics which demonstrate that a trial that applies an optimal data-driven set of EC will better represent the patient population who will receive the medication in routine clinical care. The restrictiveness of EC has been discussed widely in the literature and across different therapeutic areas (1, 3–6). Calculation of representativeness metrics such as GIST 2.0 may aid the communication of improvements in trial population attributes (sample size and representativeness) due to using data-driven EC, thereby directly addressing the concerns of the restrictiveness of clinical trial EC. However, relaxing EC raises practical implications for trial execution, such as whether to implement key EC that could not be emulated, or regulatory expectations that certain EC are clinically necessary (e.g., due to safety concerns) despite emulation analyses that conclude they should be dropped.

There are two key limitations to our approach. Firstly, in any given RWE trial emulation, the key limitations are data quality and availability, both owing to RWD not collected for the purposes of research. Key variables are either not measured or not at a frequency or temporality that they would be in a designed trial setting. We were able to replicate just over half of all EC from the published protocols (16, 17); however, we failed to include a significant number of potentially clinically meaningful criteria that might have had a modifying impact on the treatment effect estimate (e.g., eosinophil counts, pulmonary function tests, cardiac arrhythmias, etc.). The absence of these variables introduces potential for unmeasured confounding. It is worth noting, however, that this is a data limitation, not a methodological one; had these variables been available, they could have been incorporated into our PS models for adjustment. To quantify the impact of their absence, we performed a robust sensitivity analysis. As reported in Table 4, the *E*-values for our final data-driven cohorts were substantial, indicating that an unmeasured confounder would need to have a strong association (greater than four- to five-fold) with both treatment and outcome to nullify our findings. This quantitative robustness is further supported by the empirical observation that our treatment effect estimates (Table 3) remained comparable to those of the original published trials. The close alignment of our AERR with the RCT results provides compelling evidence that the net effect of any unmeasured confounding from unemulated criteria was likely not substantial enough to invalidate our conclusions.

Lastly, trial emulation in RWE may be limited by other factors in research design, including the inability to pose the same research question in routine clinical care settings or potential residual biases that could not be accounted for in analyses. Prior studies have emulated randomized controlled trials in RWE with varying degrees of success due to factors other than data quality (26, 27). A further refinement of trial emulation to consider is the use of positive or negative controls to demonstrate that internal validity of the trial emulation.

## Data Availability

A commercial vendor (Optum, Inc.) owns the database containing the data used in this manuscript, which are subject to a data use agreement. These de-identified datasets are subject to their licensing and approval terms. Requests to access these datasets should be directed to NandiniAnil.Bhosale@pfizer.com.

## References

[1] Kaur M Frahm F Lu Y Ascha MS Guadamuz JS Dotan E . Broadening eligibility criteria and diversity among patients for cancer clinical trials. NEJM Evid. (2024) 3:EVIDoa2300236. doi: 10.1056/EVIDoa2300236, 38771994

[2] Harvey RD Bruinooge SS Chen L Garrett-Mayer E Rhodes W Stepanski E . Impact of broadening trial eligibility criteria for patients with advanced non-small cell lung cancer: real-world analysis of select ASCO-*friends* recommendations. Clin Cancer Res. (2021) 27:2430–4. doi: 10.1158/1078-0432.Ccr-20-3857, 33563634

[3] Kim ES Bruinooge SS Roberts S Ison G Lin NU Gore L . Broadening eligibility criteria to make clinical trials more representative: American Society of Clinical Oncology and friends of Cancer research joint research statement. J Clin Oncol. (2017) 35:3737–44. doi: 10.1200/jco.2017.73.7916, 28968170 PMC5692724

[4] Hantel A Luskin MR Khan I Warner E Patel AA Walsh TP . Use, variability, and justification of eligibility criteria for phase II and III clinical trials in acute leukemia. Haematologica. (2024) 109:1046–52. doi: 10.3324/haematol.2023.283723, 37560812 PMC10985457

[5] Chari A Romanus D Palumbo A Blazer M Farrelly E Raju A . Randomized clinical trial representativeness and outcomes in real-world patients: comparison of 6 hallmark randomized clinical trials of relapsed/refractory multiple myeloma. Clin Lymphoma Myeloma Leuk. (2020) 20:8–17.e16. doi: 10.1016/j.clml.2019.09.625, 31722839

[6] Raymond J Boisseau W Nguyen TN Darsaut TE. Understanding why restrictive trial eligibility criteria are inappropriate. Neurochirurgie. (2024) 70:101589. doi: 10.1016/j.neuchi.2024.101589, 39244816

[7] Li Q Guo Y He Z Zhang H George TJ Jr Bian J. Using real-world data to rationalize clinical trials eligibility criteria design: a case study of Alzheimer's disease trials. AMIA Annu Symp Proc. (2021) 2020:717–26. 33936446 PMC8075542

[8] Gerber DE Singh H Larkins E Ferris A Forde PM Selig W . A new approach to simplifying and harmonizing cancer clinical trials-standardizing eligibility criteria. JAMA Oncol. (2022) 8:1333–9. doi: 10.1001/jamaoncol.2022.1664, 35925576 PMC9934063

[9] Huang H Jia S Wang X Miao H Fang H He H . Quantitative evaluation of the impact of relaxing eligibility criteria on the risk-benefit profile of drugs for lung cancer based on real-world data. Thorac Cancer. (2024) 15:1187–94. doi: 10.1111/1759-7714.15269, 38576119 PMC11091778

[10] Calaprice-Whitty D Galil K Salloum W Zariv A Jimenez B. Improving clinical trial participant prescreening with artificial intelligence (AI): a comparison of the results of AI-assisted vs standard methods in 3 oncology trials. Ther Innov Regul Sci. (2020) 54:69–74. doi: 10.1007/s43441-019-00030-4, 32008227

[11] Liu R Rizzo S Whipple S Pal N Pineda AL Lu M . Evaluating eligibility criteria of oncology trials using real-world data and AI. Nature. (2021) 592:629–33. doi: 10.1038/s41586-021-03430-5, 33828294 PMC9007176

[12] Jreich R Zhang H Meng Z Wang F. Evaluating the robustness of an AI pathfinder application on eligibility criteria in multiple myeloma trials using real-world data and historical trials. J Compar Effect Res. (2024) 13:e230164. doi: 10.57264/cer-2023-0164, 38869838 PMC11225521

[13] Optum. Market Clarity: Linked EHR and Claims data. Available online at: https://www.optum.com/en/business/insights/life-sciences/page.real-world-data.market-clarity-data.html?utm_source=chatgpt.com (Accessed February 5, 2026).

[14] U.S. Food & Drug Administration. FDA'S Sentinel Initiative. Available online at: https://www.fda.gov/safety/fdas-sentinel-initiative (Accessed February 5, 2026).

[15] Regeneron. Dupixent Full Prescribing Information. Available online at: https://www.regeneron.com/downloads/dupixent_fpi.pdf (Accessed February 5, 2026).

[16] Bhatt SP Rabe KF Hanania NA Vogelmeier CF Cole J Bafadhel M . Dupilumab for COPD with type 2 inflammation indicated by eosinophil counts. N Engl J Med. (2023) 389:205–14. doi: 10.1056/NEJMoa2303951, 37272521

[17] Castro M Corren J Pavord ID Maspero J Wenzel S Rabe KF . Dupilumab efficacy and safety in moderate-to-severe uncontrolled asthma. N Engl J Med. (2018) 378:2486–96. doi: 10.1056/NEJMoa1804092, 29782217

[18] Hernán MA Wang W Leaf DE. Target trial emulation: a framework for causal inference from observational data. JAMA. (2022) 328:2446–7. doi: 10.1001/jama.2022.21383, 36508210

[19] Austin PC Stuart EA. Moving towards best practice when using inverse probability of treatment weighting (IPTW) using the propensity score to estimate causal treatment effects in observational studies. Stat Med. (2015) 34:3661–79. doi: 10.1002/sim.6607, 26238958 PMC4626409

[20] VanderWeele TJ Ding P. Sensitivity analysis in observational research: introducing the E-value. Ann Intern Med. (2017) 167:268–74. doi: 10.7326/m16-2607, 28693043

[21] Sen A Chakrabarti S Goldstein A Wang S Ryan PB Weng C. GIST 2.0: a scalable multi-trait metric for quantifying population representativeness of individual clinical studies. J Biomed Inform. (2016) 63:325–36. doi: 10.1016/j.jbi.2016.09.003, 27600407 PMC5077682

[22] Administration USFD. Diversity action Plans to Improve Enrollment of Participants from Underrepresented Populations in Clinical Studies. Available online at: https://www.fda.gov/regulatory-information/search-fda-guidance-documents/diversity-action-plans-improve-enrollment-participants-underrepresented-populations-clinical-studies (Accessed February 5, 2026).

[23] Riner AN Girma S Vudatha V Mukhopadhyay N Skoro N Gal TS . Eligibility criteria perpetuate disparities in enrollment and participation of black patients in pancreatic cancer clinical trials. J Clin Oncol. (2022) 40:2193–202. doi: 10.1200/jco.21.02492, 35316089 PMC9273372

[24] Kelsey MD Patrick-Lake B Abdulai R Broedl UC Brown A Cohn E . Inclusion and diversity in clinical trials: actionable steps to drive lasting change. Contemp Clin Trials. (2022) 116:106740. doi: 10.1016/j.cct.2022.106740, 35364292 PMC9133187

[25] Petrik AF Henrikson NB Coronado GD Keast E Banegas MP. A roadmap for improving representation in clinical trials. Contemp Clin Trials Commun. (2024) 42:101374. doi: 10.1016/j.conctc.2024.101374 39391227 PMC11466549

[26] Suissa S Schneeweiss S Feldman WB Tesfaye H Wang SV. Emulating randomized trials by observational database studies: the RCT-DUPLICATE initiative in COPD and asthma. Am J Epidemiol. (2025) 194:1152–9. doi: 10.1093/aje/kwae319, 39191649 PMC12055465

[27] Wang SV Schneeweiss S RCT-DUPLICATE Initiative. Emulation of randomized clinical trials with nonrandomized database analyses: results of 32 clinical trials. JAMA. (2023) 329:1376–85. doi: 10.1001/jama.2023.4221, 37097356 PMC10130954

[28] DeTora LM Toroser D Sykes A Vanderlinden C Plunkett FJ Lane T . Good publication practice (GPP) guidelines for company-sponsored biomedical research: 2022 update. Ann Intern Med. (2022) 175:1298–304. doi: 10.7326/m22-1460, 36037471

