# Supplementary material for: Data-driven trial design: use of target trial emulation to evaluate eligibility criteria in asthma and COPD
Source: Front Med (Lausanne). 2026 Jun 23;13:1863026. doi: 10.3389/fmed.2026.1863026 (PMC13337763; doi:10.3389/fmed.2026.1863026)
Supplement: Supplementary file 1 [file Supplementary_file_1.DOCX]

**Supplementary information**

**Technical details of methods applied**

This paper uses a novel statistical methodology and Trial Pathfinder adaptations to fit count data for the target trial outcomes of moderate/severe exacerbation rate, for asthma and chronic obstructive pulmonary disease (COPD). For emulating the Asthma/COPD trials, we demonstrate the steps for emulating and evaluating the trial eligibility criteria:

1. **Cohort selection:** The set of all emulated trial criteria is applied in real-world data (RWD) to create a patient cohort that emulates the original trial population. Not all criteria could be emulated (see Table S1 and S2), due to missing information in the Optum electronic health records.
   1. **In the Asthma trial emulation,** the requirement for physician-diagnosed asthma for ≥12 months, as per GINA 2017 guidelines, was used to prepare the analysis cohort i.e. initial cohort included only patients meeting these foundational criteria, to achieve a baseline comparability with the NCT02414854 trial population. Some criteria could not be emulated due to the unavailability of corresponding data in the RWD. For instance, pre-bronchodilator FEV1 ≤80% of predicted normal for adults and ≤90% for adolescents at Visits 1 and 2 (prior to randomization) was excluded due to a 92% missing rate for FEV1-related variables in the dataset.
   2. **In the COPD trial emulation,** the analysis cohort was restricted to patients with physician-diagnosed COPD, to achieve baseline comparability with the NCT03930732 trial population. Due to the unavailability of corresponding data in the RWD, certain criteria could not be emulated, such as post‑bronchodilator FEV1/FVC ≤70% and post-bronchodilator FEV1 % predicted between 30% and 70%, which had more than 90% missing rate in the dataset.
2. **Automated eligibility criteria encoder:** Each trial eligibility criteria were encoded as logic rules following the Trial Pathfinder workflow.
3. **Balancing covariates:** Covariate balance was achieved by adopting propensity score adjustments.
4. **Propensity score adjustments:** To emulate randomization and adjust for confounding, stabilized inverse probability of treatment weighting (IPW) was applied to estimate the annualized exacerbation rate ratio (AERR, and 95% confidence intervals) (19). The propensity score weights were used for estimating AERR by IPW and for obtaining doubly robust estimates for AERR using AIPW (augmented inverse probability weighting).

Logistic regression was used to estimate the propensity scores ($\pi_{i}$); each patient was assigned a stabilized weight ($w_{i}$​), where each $w_{i}=\frac{A_{i}}{\pi_{i}}+\frac{1-A_{i}}{1-\pi_{i}};$and $A_{i}=1$ for treated, $A_{i}=0$for control.

1. **Shapley computation and evaluation of optimal set of criteria:** The effect of each eligibility criteria is assessed in silico by Shapley values. The Shapley value for a single criterion, say $i,$ is the weighted average (difference in AERR for our case) of the effect of adding this criterion to different subsets of inclusion/exclusion criteria. The Shapley value for criteria i is defined as

$$\sum_{S\subseteq N\backslash\{i\}} \begin{aligned} \left( \frac{\left| S \right|!\left( n-\left| S \right|-1 \right)!}{n!} \right)\left( IRR\left( S\cup\left\{ i \right\} \right)-IRR\left( S \right) \right) \end{aligned}$$

Where criteria subset S is used to select patients, and n is the total number of criteria. The incidence rate ratio (IRR) for comparing treatment with control group in the above expression for Shapley value is computed for each set in the summation by propensity score weight adjusted methods (inverse probability weighting) as discussed in detail in the next step. The key point to note here is that the Shapley value for each criterion is the marginal contribution of that criterion in improving IRR. Since a lower IRR value is better, a Shapley value of less than 0 indicates that the criteria under consideration, if applied, generally improves the treatment effect. A data-driven set of criteria is determined based on all the Shapley values and the set of optimal criteria would be the ones that have Shapley value less than 0. Finally, we compared the treatment effects (based on IRR) among three different cohorts of interest:

1. Relaxed cohort: The initial cohort, without any eligibility criteria applied.
2. Original cohort: The cohort with all protocol specified criteria applied (most restrictive).
3. Data-driven cohort: The cohort with optimal set of criteria applied (Shapley value <0).
4. **Estimation of exacerbation IRR (IPW and AIPW):** Let us consider $Y$ as the outcome variable (counts of exacerbation); treatment assignment as $A$ (Treatment group = 1; Control group = 0); exposure time for subject under treatment as $E$ (maximum exposure time = 1 year) and confounders and baseline prognostics as $Z$.

Let $Y^{1}$ and $Y^{0}$ denote the potential outcomes under treatment and control respectively. The causal estimand is the AERR for asthma/COPD exacerbation comparing treatment with the control group and is given by

$$AERR=\frac{E\left( Y^{1} \right)}{E\left( Y^{0} \right)}=\frac{E\left( Y | A=1,Z \right)}{E\left( Y | A=0,Z \right)}=\frac{\Psi\left( 1 \right)}{\Psi\left( 0 \right)}$$

The IPW estimate for $\Psi\left( a \right)$, where a = 1 or 0, is

$$\hat{\Psi}_{IPW}\left( a \right)=\frac{\sum_{i} I\left( A_{i}=a \right)\times\left( y_{i}w_{i} \right)}{\sum_{i} I\left( A_{i}=a \right)\times e_{i}}$$

where $w_{i}$ is the marginally stabilized propensity score weight obtained from the logistic exposure model, stabilized by the marginal probability of each treatment group.

The estimate for AERR is given by

$$AERR_{IPW}=\frac{\hat{\Psi}_{IPW}\left( a=1 \right)}{\hat{\Psi}_{IPW}\left( a=0 \right)}$$

To further improve robustness of the final estimates of AERR in the different cohorts (with relaxed, data-driven, and original criteria applied), AIPW was applied. As a natural extension of IPW, the AIPW estimate for AERR for more robust estimation is given by

$$AERR_{AIPW}=\frac{\hat{\Psi}_{AIPW}\left( a=1 \right)}{\hat{\Psi}_{AIPW}\left( a=0 \right)}$$

and

$$\hat{\Psi}_{AIPW}\left( a \right)=\frac{\sum_{i} I\left( A_{i}=a \right)\left\{ \left( y_{i}-\hat{\lambda}\left( Y | A=a,Z \right) \right)+\hat{\lambda}\left( Y | A:=a,Z \right) \right\}\times w_{i}}{\sum_{i} I\left( A_{i}=a \right)\times e_{i}}$$

where $A:= 1$ indicate that we set all the values of treatment assignment as 1 (treated) for prediction in $\hat{\lambda}\left( Y | A:=1,Z \right)$; and $A:= 0$ indicate that we set all the values of treatment assignment as 0 (untreated) for prediction from the outcome model $\hat{\lambda}\left( Y | A:=0,Z \right)$.

1. **Sensitivity analysis using E-values:** The robustness of the AERR estimates for each cohort (with relaxed, data-driven and original criteria applied) was assessed for unmeasured confounding using E-values.

The E-value was calculated for the observed treatment effect and the corresponding confidence intervals; and they quantify the minimum strength of association (on the risk‑ratio scale) that an unmeasured confounder would need to have with both the treatment and the outcome to explain away (alter) the observed effect.

Since the estimated IRR for exacerbation $IRR$, for comparing dupilumab with control, is less than 1, we define $AERR* = 1/AERR$ and E-value is given by

$$E = \mathrm{AER}R^{*}+\sqrt{AERR^{*}\times\left( AERR^{*}-1 \right)}$$

This provides the minimum association strength needed for an unmeasured confounder to nullify the observed treatment effect.

E-value for the confidence interval bound in case $AERR$ is less than 1, and if the upper limit of the 95% CI (UL) is greater than 1; then $E = 1$. If UL <1, then we define E-value in terms of $UL* = 1/UL$ to test the sensitivity of the upper CI we use

$$E_{UL}=UL^{*}+\sqrt{UL^{*}\times\left( UL^{*}-1 \right)}$$

**Table S1 Trial eligibility criteria and RWD emulation for COPD dupilumab.**

| **Endpoint** | | **Details** | **Emulation criteria** |
| --- | --- | --- | --- |
| Moderate or severe COPD exacerbation | | Annualized rate of acute moderate or severe COPD exacerbations (AECOPD, as defined in I02) | Deterioration of COPD requiring use of systemic corticosteroids for ≥3 days for COPD, or hospitalization or emergency room visit because of COPD, requiring systemic corticosteroids for any duration |
| **Criterion** | **Inclusion criteria** | **Details** | **Emulation criteria** |
| **I01** | Age | Patient must be ≥40 to ≤80 years of age, at the time of signing the informed consent | Age on index |
| **I02** | Diagnosis | Physician diagnosis of COPD | ICD code J41-J44 in baseline period |
|  | Smoking history | Current or former smokers with a smoking history of ≥10 pack-years | Ever reported as a current smoker in baseline period |
|  | COPD severity | Moderate to severe COPD (post-bronchodilator FEV1/FVC ≤70% and post-bronchodilator FEV1 % predicted >30% and ≤70%) | ICD code J41-J44 in baseline period |
|  | Dyspnea scale | MRC dyspnea scale Grade ≥2 | ICD-10 code R06.0X in baseline period |
|  | Chronic bronchitis | Patient-reported history of signs and symptoms of chronic bronchitis (chronic productive cough) for 3 months in the year up to screening | Presence of one or more ICD-10 codes for cough in baseline period; code list in separate table |
|  | Exacerbation history | Documented history of high exacerbation risk (≥2 moderate or ≥1 severe within the year prior to inclusion). At least one exacerbation should have occurred while the patient was taking ICS/LABA/LAMA (or LABA/LAMA if ICS is contraindicated). Moderate exacerbations were recorded by the investigator and defined as AECOPD that required either systemic corticosteroids (intramuscular, intravenous, or oral) and/or antibiotics. One of the two required moderate exacerbations had to require the use of systemic corticosteroids. Severe exacerbations were recorded by the investigator and defined as AECOPD requiring hospitalization or observation >24 hours in emergency department/urgent care facility | An exacerbation was defined as a deterioration of COPD requiring use of systemic corticosteroids for ≥3 days for COPD, or hospitalization or emergency room visit because of COPD, requiring systemic corticosteroids for any duration during the baseline period |
|  | Background therapy | Triple therapy (ICS + LABA + LAMA) for 3 months prior to randomization with a stable dose of medication for ≥1 month prior to Visit 1; double therapy (LABA + LAMA) allowed if ICS was contraindicated | ICS + LAMA + LABA within 3 months prior to index. Code list under review |
| **I03** | Evidence of type 2 inflammation | Patients with blood eosinophils ≥300 cells/microliter at Visit 1 (Screening) | Eosinophilia ICD-10 code and available lab data within baseline period |
| **I04** | Weight | BMI ≥16 kg/m² | Last BMI result in baseline period, calculated as last BMI result in baseline period, calculated as weight in pounds divided by the square height (inches) with unit conversion to kg/m^2^ |
| **I05** | Pregnancy risk | Male or female. Contraceptive use by women should be consistent with local regulations regarding the methods of contraception for those participating in clinical studies. A female patient is eligible to participate if she is not pregnant, not breastfeeding, and at least one of the following conditions applies: not a WOCBP or a WOCBP who agrees to follow the contraceptive guidance during the intervention period and for at least 12 weeks after the last dose of study intervention | Not implemented |
| **I06** | Informed consent | Capable of giving signed informed consent which includes compliance with the requirements and restrictions listed in the ICF and in this protocol | Not implemented |
| **Criterion** | **Exclusion criteria** | **Details** | **Emulation criteria** |
| **E01** | COPD diagnosis duration | COPD diagnosis for less than 12 months prior to randomization | No J41-J44 codes -12 to -18 months prior to index date |
| **E02** | Asthma diagnosis | A current diagnosis of asthma according to the 2018 GINA guidelines or other accepted guidelines; or any history of asthma diagnosis ≥40 years of age | Implement 1Ip or 2Op asthma codes within baseline period |
| **E03** | Other pulmonary diseases | Significant pulmonary disease other than COPD (e.g. lung fibrosis, sarcoidosis, interstitial lung disease, pulmonary hypertension, bronchiectasis, Churg-Strauss Syndrome, etc.) or another diagnosed pulmonary or systemic disease associated with elevated peripheral eosinophil counts | Codes for significant lung diseases in baseline: asbestosis, asthma, bronchiectasis, bronchitis, chronic cough, COVID-19, cystic fibrosis, eosinophilic granulomatosis with polyangiiti, hantavirus pulmonary syndrome, influenza, ILD, long COVID, lung cancer, pertussis (whooping cough), pleurisy, pneumonia, pulmonary embolism, pulmonary fibrosis, pulmonary hypertension, pulmonary sarcoidosis, RSV, sleep apnea, SIDS, tuberculosis |
| **E04** | Cardiac conditions | Cor pulmonale, evidence of right cardiac failure | ICD code I27.81 in baseline period |
| **E05** | Oxygen therapy | Treatment with oxygen of more than 12 hours per day | CPT code 94645 in baseline period |
| **E06** | Hypercapnia | Hypercapnia requiring BiPAP | ICD10 J96. 02 in baseline period |
| **E07** | Recent exacerbation | AECOPD (as defined above in I 02) within 4 weeks prior to or during the screening period | COPD exacerbation (i.e. endpoint) 1 month prior to index |
| **E08** | Respiratory infection | Respiratory tract infection within 4 weeks prior to screening, or during the screening period | Any ICD code for respiratory tract Infections (code list attached separately) within 1 month prior to index |
| **E09** | Surgical history | History of (or planned) pneumonectomy or lung volume reduction surgery. Patients who are participating in the acute phase of a pulmonary rehabilitation program, i.e. who started rehabilitation <4 weeks prior to screening (note: patients in the maintenance phase of a rehabilitation program can be included) | CPT code 32442 or 32491 in baseline period |
| **E10** | α-1 anti-trypsin deficiency | Diagnosis of α-1 anti-trypsin deficiency | ICD-10 code E88. 01 in baseline period |
| **E11** | Language or psychological issues | Inability to follow the procedures of the study (e.g. due to language problems, psychological disorders) or unable to read, understand and fill out a questionnaire or use an e‑Diary without any help | Not implemented |
| **E12** | Biologic therapy | Anti-IgE therapy (omalizumab) within 130 days prior to Visit 1 or any other biologic therapy (including anti-IL5 mAb) or immunosuppressant to treat inflammatory disease or autoimmune disease (e.g. rheumatoid arthritis, inflammatory bowel disease, primary biliary cirrhosis, systemic lupus erythematosus, multiple sclerosis, etc.) as well as other diseases within 2 months or 5 half-lives prior to Visit 1, whichever is longer | Any NDC code for immunosuppressant therapies listed as documented in code list “immunosuppressants” |
| **E13** | Investigative drug exposure | Exposure to another investigative drug (small molecules as well as monoclonal antibodies) within a time period prior to Visit 1 that is less than 6 months. The minimum interval since exposure to any other (non-antibody) investigative study medication is 30 days prior to Visit 1 | Not implemented |
| **E14** | Hypersensitivity | History of systemic hypersensitivity or anaphylaxis to any biologic therapy, including any excipients | Not implemented |
| **E15** | Prohibited medications | Patients receiving medication or therapy that are prohibited as concomitant therapy | Not implemented |
| **E16** | Investigator or staff | Patient is the investigator, or any sub-investigator, research assistant, pharmacist, study coordinator, other staff or relative thereof directly involved in the conduct of the study | Not implemented |
| **E17** | ECG abnormalities | Clinically significant abnormal ECG at randomization that may affect the conduct of the study in the judgment of the investigator, prolonged QTc interval (male >450 msec, female >470 msec, Fredericia correction) | Not implemented |
| **E18** | Significant medical illness | A patient with a history of clinically significant renal, hepatic, cardiovascular, metabolic, neurologic, hematologic, ophthalmologic, respiratory, gastrointestinal, cerebrovascular, substance and/or alcohol abuse, prior history of malignancy or active malignancy, including lymphoproliferative diseases (except successfully-treated carcinoma in situ of the cervix, nonmetastatic squamous cell or basal cell carcinoma of the skin) within 5 years prior to baseline, or other significant medical illness or disorder which, in the judgment of the investigator, could interfere with the study or require treatment that might interfere with the study. Specific examples include but are not limited to poorly controlled insulin-dependent diabetes, uncontrolled hypertension | Not implemented |
| **E19** | Tuberculosis | Active tuberculosis or non-tuberculous mycobacterial infection, latent untreated tuberculosis or a history of incompletely treated tuberculosis will be excluded from the study unless it is well documented by a specialist that the patient has been adequately treated and can now start treatment with a biologic agent, in the medical judgment of the investigator and/or infectious disease specialist | Not implemented |
| **E20** | Recent cardiovascular events | Acute myocardial infarction <6 months from screening visit | ICD-10 I21.XX; I22.XX, I23.XX in 6 months prior to index date |
| **E21** | TIA or stroke | TIA or stroke <6 months from screening visit | ICD-10 I21.XX; I22.XX, I23.XX in 6 months prior to index date |
| **E22** | Hospitalization | Hospitalization for any CV or cerebrovascular event <6 months from screening visit | ICD-10 code for conditions listed in above CV and cerebrovascular code lists |
| **E23** | Heart failure | Heart failure NYHA Class III or IV | Not implemented |
| **E24** | Cardiac medications | Patients on cardiac medications not on a stable dose during the last 6 months, e.g. antiarrhythmics, antihypertensives, and antidiuretics, etc. Dose modification of cholesterol-modifying agents and anticoagulants is allowed | Not implemented |
| **E25** | Cardiac arrhythmias | Cardiac arrhythmias including paroxysmal (e.g. intermittent) atrial fibrillation are excluded. Patients with persistent atrial fibrillation as defined by continuous atrial fibrillation for at least 6 months and controlled with a rate control strategy (i.e. selective beta blocker, calcium channel blocker, pacemaker placement, digoxin, or ablation therapy) and stable appropriate level of anticoagulation for at least 6 months may be considered for inclusion | Not implemented |
| **E26** | Unstable ischemic heart disease | Unstable ischemic heart disease or other relevant cardiovascular disorder such as pulmonary embolism or deep vein thrombosis within ≤6 months from enrollment that in investigator's judgment may put the patient at risk or negatively affect the study outcome | Not implemented |
| **E27** | Controller therapy compliance | Patients who are <80% compliant with controller therapy during screening | Not implemented |
| **E28** | Previous dupilumab use | Previous use of dupilumab | NDC code for dupilumab in baseline period |
| **E29** | Pregnancy or lactation | Females who are lactating, breastfeeding, or who are pregnant | ICD-10 codes Z33.1 and Z39.1 for pregnancy and lactation in baseline period |
| **E30** | Contraceptive use | Women of childbearing potential (pre-menopausal female biologically capable of becoming pregnant) who do not have a confirmed negative serum beta-HCG test at Visit 1 or negative urine pregnancy test at Visit 2 OR who are not protected by one of the following acceptable forms of effective contraception during the study: established use of oral, injected or implanted or inserted hormonal contraceptive (IUD with copper or IUS with progestogen Barrier contraceptive [condom, diaphragm, or cervical vault caps] used with spermicide [foam, gel, film, cream, or suppository], if allowed by local regulation); female sterilization (e.g. tubal occlusion, hysterectomy, or bilateral salpingectomy); male sterilization with post-vasectomy documentation of the absence of sperm in the ejaculate (for female patients in the study, the vasectomized male partner should be the sole partner for that patient); or true abstinence in keeping with the preferred and usual lifestyle and if allowed by local regulation (periodic abstinence [e.g. calendar, ovulation, symptothermal, post-ovulation methods] is not an acceptable method of contraception). Postmenopausal women (defined as at least 12 consecutive months without menses) are not required to use additional contraception | NOT IMPLEMENTED |
| **E31** | Parasitic infection | Diagnosed active parasitic infection (helminthes), suspected or high risk of parasitic infection, unless clinical and (if necessary) laboratory assessments have ruled out active infection before randomization | ICD-10 code B83.9 for helminths in baseline period |
| **E32** | HIV infection | History of HIV infection or positive HIV 1/2 serology at Visit 1 | ICD-10 code B20 for HIV in baseline period |
| **E33** | Immunosuppression | Known or suspected history of immunosuppression, including history of invasive opportunistic infections (e.g. tuberculosis, histoplasmosis, listeriosis, coccidioidomycosis, penumocystosis, or aspergillosis), despite infection resolution; or unusually frequent, recurrent or prolonged infections, per investigator’s judgment | Not implemented |
| **E34** | Acute or chronic infection | Evidence of acute or chronic infection requiring treatment with antibacterials, antivirals, antifungals, antiparasitics, or antiprotozoals within 4 weeks before Visit 1, significant viral infections within 4 weeks before Visit 1 that may not have received antiviral treatment (e.g. influenza receiving only symptomatic treatment) | Any NDC code for antibacterials, antivirals, antifungals, antiparasitics, or antiprotozoals in baseline period |
| **E35** | Live vaccinations | Live, attenuated vaccinations within 4 weeks prior to Visit 1 or planned live, attenuated vaccinations during the study | Any NDC code for agents in baseline period |
| **E36** | Autoimmune disease | Patients with active autoimmune disease or patients using immunosuppressive therapy for autoimmune disease (e.g. inflammatory bowel disease, primary biliary cirrhosis, systemic lupus erythematosus, multiple sclerosis, etc.) | Any NDC code for macrolide agents in baseline period |
| **E37** | Hepatitis | Patients with any of the following result at screening: positive (or indeterminate) HBsAg or positive IgM HBc Ab or positive total HBc Ab confirmed by positive HBV DNA or positive HCV Ab confirmed by positive HCV RNA | ICD-10 code for HBV B16.XX, B17.0, B18.0, B18.1, B19.10 or HCV B17.1, B18.2, B19.2 in baseline period |
| **E38** | Laboratory abnormalities | Clinically significant laboratory tests at screening/randomization (Visit 1) including ALT >3 times the ULN, hemoglobin <10g /100 mL for male and < 9g/ 100 mL for female, platelets <100 000/mm3, or creatinine ≥150 μmol/L | Apply if lab value exists 6 months prior to index date |
| **E39** | Macrolide therapy | Patients on macrolide (e.g. azithromycin) therapy, unless on stable therapy for >12 months | Any NDC code for macrolide agents in baseline period |
| **E40** | Consent withdrawal | Patient who has withdrawn consent before enrollment/randomization | Not implemented |
| **E41** | Study enrollment stopped | Despite screening of the patient, enrollment/randomization is stopped at the study level | Not implemented |

AECOPD, annualized rate of acute moderate or severe COPD exacerbation; ALT, alanine transaminase; BMI, body mass index; BiPAP, Bilevel positive airway pressure; COPD, chronic obstructive pulmonary disease; COVID-19, coronavirus 2019; CPT, current procedural terminology; CV, cardiovascular;
DNA, deoxyribonucleic acid; ECG, electrocardiogram; FEV_1_, forced expiratory volume in 1 second; FVC, forced vital capacity; GINA, global initiative for asthma; HBc Ab, hepatitis B core antibody; HBsAg, hepatitis B surface antigen; HBV, hepatitis B virus; HCG, human chorionic gonadotrophin; HCV, hepatitis C virus; HCV Ab, hepatitis C virus antibody; HIV, human immunodeficiency virus; ICD, international classification of diseases; ICF, informed consent form;
ICS, inhaled corticosteroid; IgE, immunoglobulin E; IgM, immunoglobulin M; ILD, interstitial lung disease; IUD, intrauterine device; IUS, intrauterine system; LABA, long acting beta-agonist; LAMA, long acting muscarinic agonist; mAb, monoclonal antibody; MRC, medical research council; NDC, national drug code; NYHA, New York Heart Association; QTc, corrected QT interval; RNA, ribonucleic acid; RSV, respiratory syncytial virus; SIDS, sudden infant death syndrome; TIA, transient ischemic attack; ULN, upper limit of normal range; WOCBP, woman of childbearing potential. **TABLE S2 Trial eligibility criteria and RWD emulation for asthma dupilumab.**

| **Endpoint** | | **Details** | **Emulation criteria** |
| --- | --- | --- | --- |
| Severe asthma exacerbation | | Annualized rate of severe asthma exacerbation events, defined as a deterioration of asthma that results in emergency treatment, hospitalization due to asthma, or treatment with systemic steroids | Deterioration of asthma requiring use of systemic corticosteroids for ≥3 days; or hospitalization or emergency room visit because of asthma, requiring systemic corticosteroids |
| **Criterion** | **Inclusion criteria** | **Details** | **Emulation criteria** |
| **I01** | Age | Adults and adolescent patients | Age ≥12 |
|  | Asthma | Physician diagnosis of asthma for ≥12 months, based on the GINA 2014 guidelines | ICD-10 code for asthma |
|  | Existing treatment | Existing treatment with medium to high dose ICS (≥500 mcg of fluticasone propionate twice daily or equivalent) in combination with a second controller (e.g. LABA, LTRA) for at least 3 months with a stable dose for ≥1 month prior to Visit 1. Patients requiring a third controller for their asthma will be considered eligible for this study, and it should also be used for at least 3 months with a stable dose for ≥1 month prior to Visit 1 | 1) Patients prescribed or administered any of the LABA-ICS-Combinations medication codes during the baseline period 2) Patients with overlapping treated days for an ICS rx code (route has to be “Inhalation”) and LABA or LTRA medication codes during the baseline period |
|  | Pre-bronchodilator FEV1 | Pre-bronchodilator FEV_1_ ≤80% of predicted normal for adults and ≤90% of predicted normal for adolescents at Visits 1 and 2, prior to randomization | Not implemented |
|  | ACQ-5 score | ACQ-5 score ≥1.5 at Visits 1 and 2, prior to randomization | Not implemented |
|  | Reversibility | Reversibility of at least 12% and 200 mL in FEV_1_ after the administration of 200 to 400 mcg albuterol/salbutamol or levalbuterol/levosalbutamol (2 to 4 inhalations of albuterol/salbutamol or levalbuterol/levosalbutamol, or of a nebulized solution of albuterol/salbutamol or levalbuterol/levosalbutamol, if considered as a standard office practice) before randomization | Not implemented |
|  | Asthma exacerbation history | Must have experienced, within 1 year prior to Visit 1, any of the following events: treatment with a systemic steroid (oral or parenteral) for worsening asthma at least once; hospitalization or emergency medical care visit for worsening asthma | 1) Any systemic corticosteroid use during the baseline period 2) An inpatient or emergency department encounter with a J45* diagnosis code during the baseline period |
| **I02** | Informed consent | Signed written informed consent | Not implemented |
| **Criterion** | **Exclusion criteria** | **Details** | **Emulation criteria** |
| **E01** | Age | Patients <12 years of age or the minimum legal age for adolescents in the country of the investigative site, whichever is higher (for those countries where local regulations permit enrollment of adults only, patient recruitment will be restricted to those who are ≥18 years of age) | Age <12 |
| **E02** | Weight | Weight is less than 30 kilograms | Weight <30 kg/<66.1387 lbs |
| **E03** | Lung diseases | COPD or other lung diseases (e.g. idiopathic pulmonary fibrosis, Churg-Strauss Syndrome, etc.) which may impair lung function | ICD-10 codes for conditions in "significant lung disease" code list |
| **E04** | Severe asthma exacerbation | A patient who experiences a severe asthma exacerbation (defined as a deterioration of asthma that results in emergency treatment, hospitalization due to asthma, or treatment with systemic steroids) at any time from 1 month prior to the screening visit up to and including the baseline visit | Deterioration of asthma requiring use of systemic corticosteroids for ≥3 days, or hospitalization or emergency room visit because of asthma, requiring systemic corticosteroids within 1 month prior to the index date |
| **E05** | Lung disease evidence | Evidence of lung disease(s) other than asthma, either clinical evidence or imaging (Chest X-ray, CT, MRI) within 12 months of Visit 1 or at the screening visit, as per local standard of care | ICD-10 codes for conditions in "significant lung disease" code list |
| **E06** | Respiratory infection | A patient who has experienced an upper or lower respiratory tract infection within the 4 weeks prior to Visit 1 or during the screening period | ICD-10 codes for conditions in "upper respiratory tract infection" code list within 4 weeks of index date |
| **E07** | Smoking status | Current smoker or cessation of smoking within 6 months prior to Visit 1 | Current smoker in Optum |
| **E08** | Smoking history | Previous smoker with a smoking history >10 pack‑years | Baseline pack-years >10 |
| **E09** | Comorbid disease | Comorbid disease that might interfere with the evaluation of IMP | Not implemented |
| **E10** | Alcohol/drug abuse | Known or suspected alcohol and/or drug abuse | Alcohol use |
| **E11** | Study procedures | Inability to follow the procedures of the study (e.g. due to language problems or psychological disorders) | Not implemented |
| **E12** | Beta-1 adrenergic receptor blockers | Patients requiring non-selective beta-1 adrenergic receptor blockers for any reason, or initiation or change in dose of a selective beta-1 adrenergic receptor blocker within 1 month prior to Visit 1 or plan to initiate or change in dose of a selective beta‑1 adrenergic receptor blocker during the screening period or the randomized treatment period | Any NDC for non-selective beta-1 adrenergic receptor blockers in baseline period |
| **E13** | Anti-IgE therapy | Anti-IgE therapy (omalizumab) within 130 days prior to Visit 1 or any other biologic therapy/immunosuppressant to treat inflammatory disease or autoimmune disease (e.g. rheumatoid arthritis, inflammatory bowel disease, primary biliary cirrhosis, systemic lupus erythematosus, multiple sclerosis, etc.) as well as other diseases within 2 months or 5 half-lives prior to Visit 1, whichever is longer | Any NDC code for immunosuppressant therapies listed as documented in code list “immunosuppressants” |
| **E14** | Allergen immunotherapy | Initiation of allergen immunotherapy within 3 months prior to Visit 1 or dose change from 1 month prior to Visit 1 or a plan to begin allergen immunotherapy or to change its dose during the screening period or the randomized treatment period | Any NDC codes for Pollinex R, Grastek, Ragwitek, Oralair |
| **E15** | Bronchial thermoplasty | Patients on or initiation of bronchial thermoplasty within 3 years prior to Visit 1 or plan to begin therapy during the screening period or the randomized treatment period | CPT code 31661, 31660 in baseline period |
| **E16** | Investigative antibody | Exposure to another investigative antibody within a time period prior to Visit 1 that is less than 5 half‑lives of the antibody. In case the half-life is not known, then the minimum interval since exposure to the prior investigative antibody is 6 months. The minimum interval since exposure to any other (non‑antibody) investigative study medication is 30 days prior to Visit 1 | Not implemented |
| **E17** | Prohibited medications | Patients receiving medications or therapy that are prohibited as concomitant medications | Any NDC codes for prohibited medications |
| **E18** | Previous dupilumab treatment | Patients who have previously been treated in any clinical trial of dupilumab | Not implemented |
| **E19** | Investigator or staff | Patient is the investigator or any sub-investigator, research assistant, pharmacist, study coordinator, other staff or relative thereof directly involved in the conduct of the study | Not implemented |
| **E20** | Non-compliance | Non-compliance with use of the mandatory background therapy, e.g. ICS/LABA combination, during the screening period, as defined as: <80% of total number of prescribed doses of background medication taken during the screening period. Compliance is verified based on background medication use recorded on the patient electronic diary during the screening period | Not implemented |
| **E21** | Significant medical illness | A patient with a history of clinically significant renal, hepatic, cardiovascular, metabolic, neurologic, hematologic, ophthalmologic, respiratory, gastrointestinal, cerebrovascular, or other significant medical illness or disorder which, in the judgment of the investigator, could interfere with the study or require treatment that might interfere with the study. Specific examples include but are not limited to uncontrolled diabetes, uncontrolled hypertension, and bronchiectasis. Active tuberculosis or non‑tuberculous mycobacterial infection, latent untreated tuberculosis, or a history of incompletely treated tuberculosis will be excluded from the study unless it is well documented by a specialist that the patient has been adequately treated and can now start treatment with a biologic agent, in the medical judgment of the investigator and/or infectious disease specialist. Tuberculosis testing would be performed on a country-by-country basis according to local guidelines if required by regulatory authorities or ethic committees. Other conditions that are well controlled and stable will not prohibit participation if deemed appropriate per the investigator’s judgment | Not implemented |
| **E22** | Pregnancy/breastfeeding | Pregnant or breastfeeding women | ICD-10 codes Z33.1 and Z39.1 for pregnancy and lactation in baseline period |
| **E23** | Women of childbearing potential | Women of childbearing potential (pre-menopausal female biologically capable of becoming pregnant) who do not have a confirmed negative serum beta‑HCG test at Visit 1 and who are not protected by one of the following acceptable forms of effective contraception during the study: established use of oral, injected, implanted, or inserted hormonal contraceptive (IUD with copper or IUS with progestogen) or barrier contraceptive (condom, diaphragm or cervical/vault caps) used with spermicide (foam, gel, film, cream, or suppository), if allowed by local regulation; female sterilization (e.g. tubal occlusion, hysterectomy, or bilateral salpingectomy); male sterilization with post‑vasectomy documentation of the absence of sperm in the ejaculate (for female patients the study, the vasectomized male partner should be the sole partner for that patient); or true abstinence in keeping with the preferred and usual lifestyle and if allowed by local regulation (periodic abstinence [e.g. calendar, ovulation, symptothermal, post‑ovulation methods] is not an acceptable method of contraception). Postmenopausal women (defined as at least 12 consecutive months without menses) are not required to use additional contraception | Not implemented |
| **E24** | Deleted | Deleted | Not implemented |
| **E25** | Parasitic infection | Diagnosed active parasitic infection (helminths), suspected or high risk of parasitic infection, unless clinical and (if necessary) laboratory assessments have ruled out active infection before randomization | ICD-10 code B83.9 for helminths in baseline period |
| **E26** | HIV infection | History of HIV infection or positive HIV serology at Visit 1 | ICD-10 code B20 for HIV in baseline period |
| **E27** | Immunosuppression | Known or suspected history of immunosuppression, including history of invasive opportunistic infections (e.g. histoplasmosis, listeriosis, coccidioidomycosis, pneumocystis, aspergillosis), despite infection resolution; or unusually frequent, recurrent or prolonged infections, per investigator’s judgment | Not implemented |
| **E28** | Acute/chronic infection | Evidence of acute or chronic infection requiring systemic treatment with antibacterials, antivirals, antifungals, antiparasitics, or antiprotozoals within 4 weeks before Visit 1 or during the screening period, significant viral infections within 4 weeks before Visit 1 or during the screening period that may not have received antiviral treatment (e.g. influenza receiving only symptomatic treatment) | Any NDC code for antibacterials, antivirals, antifungals, antiparasitics, or antiprotozoals in baseline period |
| **E29** | Live attenuated vaccinations | Live attenuated vaccinations within 4 weeks prior to Visit 1 or planned live attenuated vaccinations during the study | Any NDC code for agents |
| **E30** | Autoimmune disease | Patients with active autoimmune disease or patients using immunosuppressive therapy for autoimmune disease (e.g. rheumatoid arthritis, inflammatory bowel disease, primary biliary cirrhosis, systemic lupus erythematosus, multiple sclerosis, etc.) or patients with high titer autoantibodies at screening who are suspected of having high risk for developing autoimmune disease at the discretion of the investigator or the sponsor | Any NDC code for immunosuppressant therapies listed as documented in code list “immunosuppressants” |
| **E31** | History of malignancy | History of malignancy within 5 years before the screening visit, except completely treated in situ carcinoma of the cervix, completely treated and resolved nonmetastatic squamous or basal cell carcinoma of the skin | Any malignancy codes as documented in tab "cancer codes" |
| **E32** | Hypersensitivity | Patients with a history of a systemic hypersensitivity reaction, other than localized injection site reaction, to any biologic drug | Not implemented |
| **E33** | Hepatitis screening | Patients with any of the following result at screening: positive (or indeterminate) HBsAg or positive IgM HBc Ab or positive total HBc Ab confirmed by positive HBV DNA or positive HCV Ab confirmed by positive HCV RNA | ICD-10 code for HBV B16.XX, B17.0, B18.0, B18.1, B19.10 or HCV B17.1, B18.2, B19.2 in baseline period |
| **E34** | Liver injury | Clinically significant/active hepatobiliary disease or ALT >3 ULN | ICD-10 code S36.11 or any ALT lab within 6 months of index >3xULN |
| **E35** | Abnormal lab values | Abnormal lab values at screening: CPK >10 ULN or platelets <100,000 cells/mm3 or eosinophils >1500 cells/mm3 | Within 6 months of index date CPK >10 ULN or platelets <100,000 cells/mm3 or eosinophils >1500 cells/mm3 |
| **E36** | Consent withdrawal | Patient who has withdrawn consent before enrollment/randomization | Not implemented |

ACQ-5, asthma control questionnaire 5-question version; ALT, alanine aminotransferase; COPD, chronic obstructive pulmonary disease; CPK, creatine phosphokinase; CPT; current procedural terminology; CT, computed tomography; DNA, deoxyribonucleic acid; FEV_1_, forced expiratory volume in 1 second; GINA, global initiative for asthma; HBc Ab, hepatitis B core antibody; HBsAg, hepatitis B surface antigen; HBV, hepatitis B virus; HCV, hepatitis C virus;
HCV Ab, hepatitis C virus antibody; HIV, human immunodeficiency virus; ICD, international classification of diseases; ICS, inhaled corticosteroid;
IgE, immunoglobulin E; IgM, immunoglobulin M; IMP, investigational medicinal product; IUD, intrauterine device; IUS, intrauterine system; LABA, long acting beta-agonist; LTRA, leukotriene receptor antagonist; MRI, magnetic resonance imaging; NDC, national drug code; RNA, ribonucleic acid; ULN, upper limit of normal range.

**TABLE S3 Propensity score model variables.**

| **COPD** | **Asthma** |
| --- | --- |
| COPD diagnosis duration | Asthma exacerbation (1 month pre-index) |
| Previous dupilumab use | Asthma exacerbation history |
| Asthma diagnosis | Abnormal lab values |
| Cardiac conditions | Live vaccination (1 month prior) |
| Smoking history | Hepatobiliary disease/ALTE36 |
| Biologic therapy | Beta blockers (non-selective) |
| Recent cardiovascular events | HIV |
| α-1 anti-trypsin deficiency | Smoking status |
| BMI | Alcohol use |
| Surgical history | Parasitic infection |
| Macrolide therapy | Allergen immunotherapy (3 months prior) |
| Parasitic infection | Bronchial thermoplasty |
| Hospitalization | Hepatitis screening |
| Live vaccinations | RTI (1 month prior) |
| Recent exacerbation | Weight |
| Oxygen therapy | Lung disease |
| Recent cardiovascular events 2 | Pregnancy |
| TIA or stroke | Malignancy (excluding BCC) |
| Pregnancy or lactation | Prohibited concomitant therapy |
| Respiratory infection | Age |
| HIV infection | Infection |
| Autoimmune disease | Race |
| Laboratory abnormalities | Sex |
| Hypercapnia | Ethnicity |
| Hepatitis |  |
| Age |  |
| Other pulmonary diseases |  |
| Acute or chronic infection |  |
| Exacerbation history |  |
| Race |  |
| Sex |  |
| Ethnicity |  |

For details of each variable, please refer to Tables S1 and S2.

ALTE36, alanine transaminase equals 36 units/liter; BCC, basal cell carcinoma; BMI, body mass index; COPD, chronic obstructive pulmonary disease; HIV, human immunodeficiency virus; RTI, respiratory tract infection; TIA, transient ischemic attack.

**TABLE S4 Analysis for asthma cohort with each criteria applied marginally.**

| **Criteria applied marginally** | **Original** | | **Fully relaxed** | | **Data-driven** | | **Data-driven criteria** |
| --- | --- | --- | --- | --- | --- | --- | --- |
|  | **AERR** | ***N*** | **AERR** | ***N*** | **AERR** | ***N*** |  |
| Age | 0.48 (0.44, 0.52) | 115,761 | 0.63 (0.61, 0.66) | 818,430 | 0.45 (0.43, 0.47) | 199,262 | Live vaccination (1 month prior), asthma exacerbation (1 month pre-index), asthma exacerbation history, smoking status |
| Weight | 0.48 (0.44, 0.52) | 115,761 | 0.68 (0.59, 0.76) | 851,282 | 0.49 (0.43, 0.54) | 664,731 | HIV, infection, live vaccination (1 month prior), malignancy (Excl. BCC), hepatitis screening, hepatobiliary disease/ALTE36, abnormal lab values |
| Lung disease at baseline | 0.48 (0.44, 0.52) | 115,761 | 0.58 (0.43, 0.74) | 809,890 | 0.43 (0.37, 0.49) | 205,823 | Abnormal lab values, beta blockers (non-selective), hepatobiliary disease/ALTE36, live vaccination (1 month prior), asthma exacerbation (1 month pre-index), asthma exacerbation history, smoking status |
| Asthma exacerbation (1 month pre-index) | 0.48 (0.44, 0.52) | 115,761 | 0.50 (0.40, 0.61) | 885,372 | 0.43 (0.32, 0.54) | 208,799 | Parasitic infection, live vaccination (1 month prior), hepatitis screening, abnormal lab values, smoking status, lung disease, asthma exacerbation history |
| Asthma exacerbation history | 0.48 (0.44, 0.52) | 115,761 | 0.57 (0.47, 0.67) | 384,559 | 0.45 (0.33, 0.57) | 226,073 | Beta blockers (non-selective), HIV, hepatitis screening, abnormal lab values, live vaccination (1 month prior), smoking status, asthma exacerbation (1 month pre-index) |
| RTI (1 month prior) | 0.48 (0.44, 0.52) | 115,761 | 0.60 (0.48, 0.74) | 867,450 | 0.44 (0.32, 0.60) | 214,328 | HIV, parasitic infection, abnormal lab values, live vaccination (1 month prior), smoking status, asthma exacerbation (1 month pre-index), asthma exacerbation history |
| Smoking status | 0.48 (0.44, 0.52) | 115,761 | 0.58 (0.46, 0.74) | 618,346 | 0.44 (0.33, 0.54) | 223,839 | Parasitic infection, hepatobiliary disease/ALTE36, hepatitis screening, abnormal lab values, live vaccination (1 month prior), asthma exacerbation (1 month pre-index), asthma exacerbation history |
| Alcohol use | 0.48 (0.44, 0.52) | 115,761 | 0.61 (0.49, 0.74) | 890,426 | 0.43 (0.31, 0.59) | 222,859 | Parasitic infection, abnormal lab values, HIV, beta blockers (non-selective), live vaccination (1 month prior), smoking status, asthma exacerbation (1 month pre-index), asthma exacerbation history |
| Beta blockers (non-selective) | 0.48 (0.44, 0.52) | 115,761 | 0.60 (0.49, 0.73) | 912,460 | 0.43 (0.31, 0.59) | 223,517 | Alcohol use, abnormal lab values, live vaccination (1 month prior), smoking status, asthma exacerbation (1 month pre-index), asthma exacerbation history |
| Allergen immunotherapy (3 month prior) | 0.48 (0.44, 0.52) | 115,761 | 0.60 (0.49, 0.74) | 915,112 | 0.43 (0.31, 0.59) | 224,179 | Parasitic infection, alcohol use, abnormal lab values, live vaccination (1 month prior), smoking status, asthma exacerbation (1 month pre-index), asthma exacerbation history |
| Bronchial thermoplasty | 0.48 (0.44, 0.52) | 115,761 | 0.61 (0.49, 0.76) | 878,523 | 0.45 (0.32, 0.57) | 213,006 | Parasitic infection, alcohol use, abnormal lab values, live vaccination (1 month prior), smoking status, asthma exacerbation (1 month pre-index), asthma exacerbation history |
| Prohibited concomitant therapy | 0.48 (0.44, 0.52) | 115,761 | 0.61 (0.49, 0.76) | 878,523 | 0.46 (0.35, 0.57) | 216,162 | Bronchial thermoplasty, hepatitis screening, abnormal lab values, smoking status, asthma exacerbation history, asthma exacerbation (1 month pre-index) |
| Pregnancy | 0.48 (0.44, 0.52) | 115,761 | 0.60 (0.49, 0.74) | 864,848 | 0.43 (0.31, 0.58) | 216,600 | Parasitic infection, bronchial thermoplasty, beta blockers (non-selective), live vaccination (1 month prior), smoking status, asthma exacerbation (1 month pre-index), asthma exacerbation history |
| Parasitic infection | 0.48 (0.44, 0.52) | 115,761 | 0.60 (0.49, 0.74) | 915,072 | 0.43 (0.31, 0.59) | 228,271 | Abnormal lab values, live vaccination (1 month prior), smoking status, asthma exacerbation history, asthma exacerbation (1 month pre-index) |
| HIV | 0.48 (0.44, 0.52) | 115,761 | 0.60 (0.49, 0.74) | 911,612 | 0.43 (0.31, 0.59) | 226,877 | Parasitic infection, abnormal lab values, beta blockers (non-selective), live vaccination (1 month prior), smoking status, asthma exacerbation (1 month pre-index), asthma exacerbation history |
| Infection | 0.48 (0.44, 0.52) | 115,761 | 0.63 (0.50, 0.79) | 772,750 | 0.45 (0.32, 0.64) | 181,863 | Hepatitis screening, abnormal lab values, hepatobiliary disease/ALTE36, smoking status, live vaccination (1 month prior), beta blockers (non-selective), asthma exacerbation history, asthma exacerbation (1 month pre-index) |
| Live vaccination (1 month prior) | 0.48 (0.44, 0.52) | 115,761 | 0.60 (0.49, 0.73) | 913,538 | 0.43 (0.31, 0.59) | 219,438 | Parasitic infection, hepatobiliary disease/ALTE36, hepatitis screening, beta blockers (non-selective), alcohol use, abnormal lab values, smoking status, asthma exacerbation history, asthma exacerbation (1 month pre-index) |
| Malignancy (Excl. BCC) | 0.48 (0.44, 0.52) | 115,761 | 0.61 (0.50, 0.75) | 863,308 | 0.43 (0.32, 0.60) | 213,503 | Parasitic infection, HIV, live vaccination (1 month prior), abnormal lab values, hepatitis screening, beta blockers (non-selective), smoking status, asthma exacerbation history, asthma exacerbation (1 month pre-index) |
| Hepatitis screening | 0.48 (0.44, 0.52) | 115,761 | 0.60 (0.49, 0.74) | 908,602 | 0.44 (0.32, 0.60) | 210,417 | Parasitic infection, RTI (1 month prior), alcohol use, abnormal lab values, beta blockers (non-selective), smoking status, asthma exacerbation history, asthma exacerbation (1 month pre-index) |
| Hepatobiliary disease/ALTE36 | 0.48 (0.44, 0.52) | 115,761 | 0.60 (0.49, 0.73) | 904,730 | 0.42 (0.29, 0.60) | 212,326 | Bronchial thermoplasty, beta blockers (non-selective), smoking status, asthma exacerbation history, asthma exacerbation (1 month pre-index) |
| Abnormal lab values | 0.48 (0.44, 0.52) | 115,761 | 0.60 (0.49, 0.74) | 903,320 | 0.40 (0.27, 0.61) | 195,242 | Prohibited concomitant therapy, parasitic infection, live vaccination (1 month prior), beta blockers (non-selective), smoking status, lung disease, asthma exacerbation history, asthma exacerbation (1 month pre-index), hepatobiliary disease/ALTE36, HIV, bronchial thermoplasty |

AERR, annualized exacerbation rate ratio; ALTE36, alanine transaminase equals 36 units/liter; BCC, basal cell carcinoma; HIV, human immunodeficiency virus; *N*, number of patients; RTI, respiratory tract infection.

**TABLE S5 Analysis for COPD cohort with each criteria applied marginally.**

| **Criteria applied marginally** | **Original** | | **Fully relaxed** | | **Data-driven** | | **Data-driven criteria** |
| --- | --- | --- | --- | --- | --- | --- | --- |
|  | **AERR** | ***N*** | **AERR** | ***N*** | **AERR** | ***N*** |  |
| COPD diagnosis duration | 0.51 (0.24, 0.79) | 4,333 | 0.46 (0.43, 0.48) | 220,727 | 0.37 (0.26, 0.49) | 96,707 | Surgical history, oxygen therapy, recent cardiovascular events2, TIA or stroke, live vaccinations, hepatitis, pregnancy or lactation, recent cardiovascular events, asthma diagnosis, previous dupilumab use, cardiac conditions, smoking history, recent exacerbation |
| Previous dupilumab use | 0.51 (0.24, 0.79) | 4,333 | 0.65 (0.63, 0.67) | 387,857 | 0.38 (0.24, 0.52) | 118,869 | Surgical history, parasitic infection, live vaccinations, BMI, recent cardiovascular events, biologic therapy, cardiac conditions, asthma diagnosis, smoking history, COPD diagnosis duration |
| Asthma diagnosis | 0.51 (0.24, 0.79) | 4,333 | 0.59 (0.52, 0.66) | 343,667 | 0.37 (0.27, 0.47) | 48,640 | Macrolide therapy, cardiac conditions, pregnancy or lactation, other pulmonary diseases, previous dupilumab use, smoking history, COPD diagnosis duration |
| Cardiac conditions | 0.51 (0.24, 0.79) | 4,333 | 0.66 (0.61, 0.70) | 385,070 | 0.39 (0.33, 0.46) | 117,811 | Biologic therapy, recent cardiovascular events, pregnancy or lactation, previous dupilumab use, asthma diagnosis, smoking history, COPD diagnosis duration |
| Smoking history | 0.51 (0.24, 0.79) | 4,333 | 0.69 (0.63, 0.76) | 276,756 | 0.39 (0.28, 0.50) | 41,846 | Parasitic infection, recent exacerbation, cardiac conditions, TIA or stroke, α-1 anti-trypsin deficiency, pregnancy or lactation, biologic therapy, asthma diagnosis, other pulmonary diseases, previous dupilumab use, COPD diagnosis duration |
| Biologic therapy | 0.51 (0.24, 0.79) | 4,333 | 0.68 (0.62, 0.77) | 387,517 | 0.36 (0.22, 0.50) | 44,661 | Surgical history, macrolide therapy, α-1 anti-trypsin deficiency, oxygen therapy, pregnancy or lactation, other pulmonary diseases, autoimmune disease, recent cardiovascular events, cardiac conditions, previous dupilumab use, asthma diagnosis, smoking history, COPD diagnosis duration |
| Recent cardiovascular events | 0.51 (0.24, 0.79) | 4,333 | 0.68 (0.64, 0.72) | 366,173 | 0.39 (0.31, 0.47) | 117,544 | Parasitic infection, surgical history, macrolide therapy, α-1 anti-trypsin deficiency, pregnancy or lactation, biologic therapy, cardiac conditions, asthma diagnosis, previous dupilumab use, smoking history, COPD diagnosis duration |
| α-1 anti-trypsin deficiency | 0.51 (0.24, 0.79) | 4,333 | 0.66 (0.60, 0.71) | 386,951 | 0.39 (0.24, 0.53) | 116,676 | Live vaccinations, recent cardiovascular events2, pregnancy or lactation, biologic therapy, cardiac conditions, recent cardiovascular events, previous dupilumab use, asthma diagnosis, smoking history, COPD diagnosis duration |
| BMI | 0.51 (0.24, 0.79) | 4,333 | 0.67 (0.64, 0.71) | 383,505 | 0.41 (0.32, 0.50) | 116,369 | Pregnancy or lactation, cardiac conditions, recent cardiovascular events, asthma diagnosis, previous dupilumab use, smoking history, COPD diagnosis duration |
| Surgical history | 0.51 (0.24, 0.79) | 4,333 | 0.67 (0.63, 0.70) | 388,042 | 0.40 (0.30, 0.49) | 116,779 | Macrolide therapy, hospitalization, biologic therapy, α-1 anti-trypsin deficiency, recent cardiovascular events, pregnancy or lactation, cardiac conditions, asthma diagnosis, previous dupilumab use, smoking history, COPD diagnosis duration |
| Macrolide therapy | 0.51 (0.24, 0.79) | 4,333 | 0.66 (0.62, 0.70) | 387,830 | 0.41 (0.34, 0.48) | 116,078 | Surgical history, parasitic infection, BMI, biologic therapy, recent cardiovascular events, α-1 anti-trypsin deficiency, pregnancy or lactation, asthma diagnosis, cardiac conditions, previous dupilumab use, smoking history, COPD diagnosis duration |
| Parasitic infection | 0.51 (0.24, 0.79) | 4,333 | 0.67 (0.65, 0.69) | 388,011 | 0.34 (0.21, 0.47) | 47,931 | Macrolide therapy, HIV infection, other pulmonary diseases, autoimmune disease, biologic therapy, α-1 anti-trypsin deficiency, cardiac conditions, asthma diagnosis, previous dupilumab use, smoking history, COPD diagnosis duration |
| Hospitalization | 0.51 (0.24, 0.79) | 4,333 | 0.67 (0.63, 0.70) | 383,210 | 0.33 (0.24, 0.42) | 48,386 | HIV infection, recent cardiovascular events2, macrolide therapy, α-1 anti-trypsin deficiency, biologic therapy, cardiac conditions, previous dupilumab use, other pulmonary diseases, asthma diagnosis, smoking history, COPD diagnosis duration |
| Live vaccinations | 0.51 (0.24, 0.79) | 4,333 | 0.66 (0.60, 0.73) | 387,772 | 0.38 (0.27, 0.48) | 116,078 | Surgical history, parasitic infection, BMI, biologic therapy, recent cardiovascular events, α-1 anti-trypsin deficiency, pregnancy or lactation, asthma diagnosis, cardiac conditions, previous dupilumab use, smoking history, COPD diagnosis duration |
| Recent exacerbation | 0.51 (0.24, 0.79) | 4,333 | 0.50 (0.45, 0.55) | 349,971 | 0.36 (0.24, 0.48) | 104,075 | Parasitic infection, macrolide therapy, pregnancy or lactation, cardiac conditions, HIV infection, α-1 anti-trypsin deficiency, recent cardiovascular events, asthma diagnosis, previous dupilumab use, smoking history, COPD diagnosis duration |
| Oxygen therapy | 0.51 (0.24, 0.79) | 4,333 | 0.67 (0.62, 0.71) | 387,004 | 0.37 (0.30, 0.44) | 123,795 | Hospitalization, parasitic infection, recent cardiovascular events2, biologic therapy, pregnancy or lactation, cardiac conditions, asthma diagnosis, previous dupilumab use, smoking history, COPD diagnosis duration |
| Recent cardiovascular events2 | 0.51 (0.24, 0.79) | 4,333 | 0.67 (0.62, 0.72) | 384,836 | 0.41 (0.32, 0.50) | 120,814 | Parasitic infection, live vaccinations, biologic therapy, α-1 anti-trypsin deficiency, pregnancy or lactation, autoimmune disease, cardiac conditions, asthma diagnosis, previous dupilumab use, smoking history, COPD diagnosis duration |
| TIA or stroke | 0.51 (0.24, 0.79) | 4,333 | 0.65 (0.60, 0.71) | 368,378 | 0.40 (0.33, 0.47) | 118,815 | Parasitic infection, macrolide therapy, HIV infection, α-1 anti-trypsin deficiency, pregnancy or lactation, biologic therapy, cardiac conditions, asthma diagnosis, previous dupilumab use, smoking history, COPD diagnosis duration |
| Pregnancy or lactation | 0.51 (0.24, 0.79) | 4,333 | 0.66 (0.60, 0.72) | 378,058 | 0.40 (0.31, 0.49) | 120,172 | Surgical history, oxygen therapy, BMI, live vaccinations, hospitalization, hepatitis, biologic therapy, cardiac conditions, previous dupilumab use, asthma diagnosis, smoking history, COPD diagnosis duration |
| Respiratory infection | 0.51 (0.24, 0.79) | 4,333 | 0.73 (0.67, 0.80) | 302,313 | 0.47 (0.35, 0.58) | 88,723 | Biologic therapy, hospitalization, live vaccinations, cardiac conditions, recent cardiovascular events, pregnancy or lactation, asthma diagnosis, previous dupilumab use, smoking history, COPD diagnosis duration |
| HIV infection | 0.51 (0.24, 0.79) | 4,333 | 0.66 (0.63, 0.70) | 386,146 | 0.37 (0.29, 0.45) | 45,721 | Surgical history, oxygen therapy, BMI, biologic therapy, recent cardiovascular events, α-1 anti-trypsin deficiency, hospitalization, cardiac conditions, previous dupilumab use, other pulmonary diseases, asthma diagnosis, smoking history, COPD diagnosis duration |
| Autoimmune disease | 0.51 (0.24, 0.79) | 4,333 | 0.66 (0.60, 0.73) | 3,735,378 | 0.40 (0.27, 0.53) | 42,744 | Surgical history, macrolide therapy, live vaccinations, recent exacerbation, pregnancy or lactation, HIV infection, biologic therapy, cardiac conditions, previous dupilumab use, other pulmonary diseases, asthma diagnosis, smoking history, COPD diagnosis duration |
| Laboratory abnormalities | 0.51 (0.24, 0.79) | 4,333 | 0.72 (0.69, 0.77) | 316,501 | 0.40 (0.27, 0.53) | 95,676 | Recent cardiovascular events2, oxygen therapy, hospitalization, live vaccinations, recent cardiovascular events, biologic therapy, cardiac conditions, asthma diagnosis, previous dupilumab use, smoking history, COPD diagnosis duration |
| Hypercapnia | 0.51 (0.24, 0.79) | 4,333 | 0.69 (0.65, 0.70) | 373,509 | 0.46 (0.40, 0.55) | 113,443 | Surgical history, HIV infection, macrolide therapy, recent cardiovascular events, pregnancy or lactation, asthma diagnosis, smoking history, previous dupilumab use, COPD diagnosis duration |
| Hepatitis | 0.51 (0.24, 0.79) | 4,333 | 0.65 (0.59, 0.71) | 380,635 | 0.40 (0.28, 0.52) | 108,597 | macrolide therapy, TIA or stroke, α-1 anti-trypsin deficiency, HIV infection, biologic therapy, live vaccinations, pregnancy or lactation, recent cardiovascular events, cardiac conditions, previous dupilumab use, asthma diagnosis, smoking history, COPD diagnosis duration |
| Age | 0.51 (0.24, 0.79) | 4,333 | 0.72 (0.66, 0.77) | 324,513 | 0.33 (0.19, 0.48) | 42,542 | Surgical history, parasitic infection, live vaccinations, other pulmonary diseases, autoimmune disease, α-1 anti-trypsin deficiency, cardiac conditions, asthma diagnosis, previous dupilumab use, smoking history, COPD diagnosis duration |
| Other pulmonary diseases | 0.51 (0.24, 0.79) | 4,333 | 0.74 (0.65, 0.83) | 124,418 | 0.31 (0.23, 0.40) | 44,502 | Live vaccinations, autoimmune disease, recent cardiovascular events, biologic therapy, hospitalization, pregnancy or lactation, previous dupilumab use, COPD diagnosis duration, smoking history |
| Acute or chronic infection | 0.51 (0.24, 0.79) | 4,333 | 0.72 (0.65, 0.79) | 277,761 | 0.48 (0.39, 0.59) | 87,794 | Parasitic infection, macrolide therapy, oxygen therapy, biologic therapy, pregnancy or lactation, asthma diagnosis, smoking history, previous dupilumab use, COPD diagnosis duration |
| Exacerbation history | 0.51 (0.24, 0.79) | 4,333 | 0.66 (0.60, 0.72) | 123,594 | 0.48 (0.32, 0.64) | 10,824 | Biologic therapy, live vaccinations, autoimmune disease, recent cardiovascular events2, previous dupilumab use, cardiac conditions, recent cardiovascular events, other pulmonary diseases, smoking history, COPD diagnosis duration |

AERR, annualized exacerbation rate ratio; BMI, body mass index; COPD, chronic obstructive pulmonary disease; HIV, human immunodeficiency virus; *N*, number of patients; TIA, transient ischemic attack.
